# Foldable Soft Leg‐Assisted Wheel Robot

**DOI:** 10.1002/advs.202512435

**Published:** 2025-10-06

**Authors:** Seunghoon Yoo, Sohyun Kim, Joohyeon Kang, Hyunjun Park, Seokjun Lee, Youngsu Cha

**Affiliations:** ^1^ School of Electrical Engineering Korea University 145 Anam‐ro Seoul 02841 South Korea; ^2^ Department of Smart Convergence Korea University 145 Anam‐ro Seoul 02841 South Korea; ^3^ Department of Autonomous IoT Research Center Korea Electronic Technology Institute 25 Saenari‑ro Seongnam 13509 South Korea

**Keywords:** folding mechanism, leg‐wheel robot, soft robotic leg

## Abstract

Although mobile robots are expanding their capabilities to perform diverse missions for humankind, the functions of mobile robots are limited according to each type of locomotion. Hybrid mobile robots, which integrate the advantages of wheel and leg systems, emerge as a solution for robots to cross operating terrain. However, these systems also encounter inherent challenges, such as increased complexity and limited terrain adaptability. Herein, a foldable soft leg is proposed to assist the functionalities of a wheel mobile robot to traverse diverse terrain. The soft leg achieves high driving force and large deformability using the inherent compliance of foldable hinges by simple control of the motor‐wire system. A hybrid mobile robot integrating the wheels and soft robotic legs is developed, exhibits posture adjustment abilities through the expansion and contraction of the legs. A novel hybrid locomotion mechanism is introduced, allowing the robot to traverse various harsh environments. Furthermore, additional equipment leveraging the unique characteristics of the soft robotic leg is also developed, enhancing the functionality of the hybrid mobile robot. This study provides valuable guidelines for the novel design of soft robotic legs to advance their potential for dexterous leg‐wheel robots.

## Graphical abstract

Recently, soft robots garner attention for designing the robotic legs of mobile robots due to their characteristic lightness, tunability, and simple control. Here, a novel soft foldable leg capable of high stiffness and deformability is identified as an assistant for enhancing the locomotion capabilities of a wheel mobile robot.

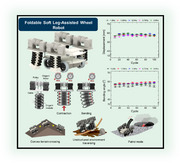

## 1 Introduction

Mobile robots are continuously expanding the boundaries of their capabilities by accomplishing missions under diverse terrestrial conditions, such as conducting search and rescue operations in the aftermath of disasters, carrying out expeditions over harsh terrain, and delivering industrial services in confined spaces.^[^ ^1^, ^2^, ^3^, ^4^ ^]^ To successfully achieve such missions, it is essential for the mobile robots to demonstrate both maneuverability and adaptability across a wide range of environments. The type of locomotion plays a pivotal role in defining the capabilities and constraints of mobile robots.^[^ ^5^, ^6^, ^7^ ^]^ Wheeled robots exhibit high speed and efficient movement on smooth surfaces but face limitations in overcoming large obstacles and rough terrain. In contrast, tracked robots are well‐suited for navigating uneven terrain and obstacles due to their extensive ground contact area, yet they inevitably encounter several challenges, including low speed, high energy consumption, and excessive vibration resulting from their discrete track profiles. Legged robots typically mimic biological organisms, harnessing effective locomotion strategies observed in animals.^[^ ^8^, ^9^, ^10^ ^]^ Although the robots excel in traversing complex environments, including structured and irregular terrain, they are prone to being structurally complex, slow, and less energy efficient compared to their counterparts.

In response to the limitations of individual locomotion types, hybrid locomotion systems with multiple movement modalities have garnered significant attention, providing a path toward versatile mobile robotic platforms.^[^ ^3^, ^5^, ^11^, ^12^, ^13^ ^]^ Among the systems, leg‐wheel robots have been developed by integrating the wheels with legs connected to the robot body.^[^ ^11^, ^12^, ^13^, ^14^, ^15^, ^16^ ^]^ The robots have achieved astonishing advancements in maneuverability, leveraging the advantages of both wheel and leg locomotion. For instance, the robots can travel across flat and compact surfaces at relatively high speeds by driving the wheels, while also overcoming the obstacle‐ridden environments using quadrupedal locomotion. Furthermore, certain leg‐wheel robots adopt retractable legs as an integral aspect of their design architecture.^[^ ^17^, ^18^, ^19^ ^]^ This mechanism enables the robot to minimize its spatial footprint when the legs are not engaged in the robot operation, thereby preventing potential interference with wheeled locomotion and unintended contact of the legs with the surrounding environment. In addition, the use of retractable legs allows for rapid deployment in response to increased terrain complexity, facilitating seamless transitions between locomotion modes and improving environmental adaptability.^[^ ^20^ ^]^ However, the retractable legs suffer from structural challenges in the realm of the leg‐wheel mechanism. Although the leg mechanism integrated into the hybrid robots significantly improves the adaptability on terrain, it inevitably results in elevated control demands by integrating leg‐wheel system to meet the obstacle crossing capabilities and a significant weight increase due to the attachment of multiple legs. Furthermore, the design requirement for the legs to support the weight of the robot while enabling structural reconfiguration for retraction and deployment can lead to increased mechanical complexity of the robot.

Recently, robotic leg structures have gained substantial momentum, driven by increasing interest in soft robots as a viable solution to key mechanical limitations.^[^ ^21^, ^22^, ^23^, ^24^ ^]^ The legs are characterized by their exceptional ability to undergo significant shape deformation with simple control.^[^ ^25^, ^26^, ^27^, ^28^, ^29^ ^]^ This capability enhances their flexibility and adaptability, particularly in response to variable and uncertain terrain conditions. Moreover, their inherent lightweight nature and superior shock absorption due to their soft base materials are advantageous for reducing the overall weight of the robotic legs and mechanical stress during locomotion.^[^ ^30^, ^31^, ^32^, ^33^ ^]^ In this context, foldable structures have emerged as a noteworthy design concept in the development of soft robotic legs.^[^ ^34^, ^35^, ^36^, ^37^, ^38^, ^39^, ^40^, ^41^ ^]^ One of the unique features of foldable structures is their ability to transform a 2D sheet into complex 3D structures through predefined crease patterns.^[^ ^42^, ^43^, ^44^ ^]^ Drastic deformation is achieved through the large strain behavior of the foldable structures, which can be particularly advantageous for enabling retractable leg mechanisms to minimize leg volume during the non‐activating phase.^[^ ^45^, ^46^, ^47^ ^]^ Moreover, by precisely engineering the folding patterns, the mechanical properties of the structure, such as directionality and force response, can be tailored. In particular, the restoring force that opposes displacement and drives a system back to equilibrium, generated by the tendency of the folded creases to return to their initial state, can be effectively harnessed for robotic applications that require lightweight yet high‐output actuation. That is, the phenomenon enables the production of instantaneous forces that are relatively large compared to the weight and size of the structure.^[^ ^48^ ^]^ By incorporating the foldable structure design concept into the main frame of the robotic legs, the natural restoration can be utilized to support the body weight of the robots and enhance their adaptability to unstructured terrain through passive deformation upon contact. Soft foldable structure, therefore, can open up new opportunities for advancing the capabilities of the leg‐wheel robot systems.

**Figure 1:**
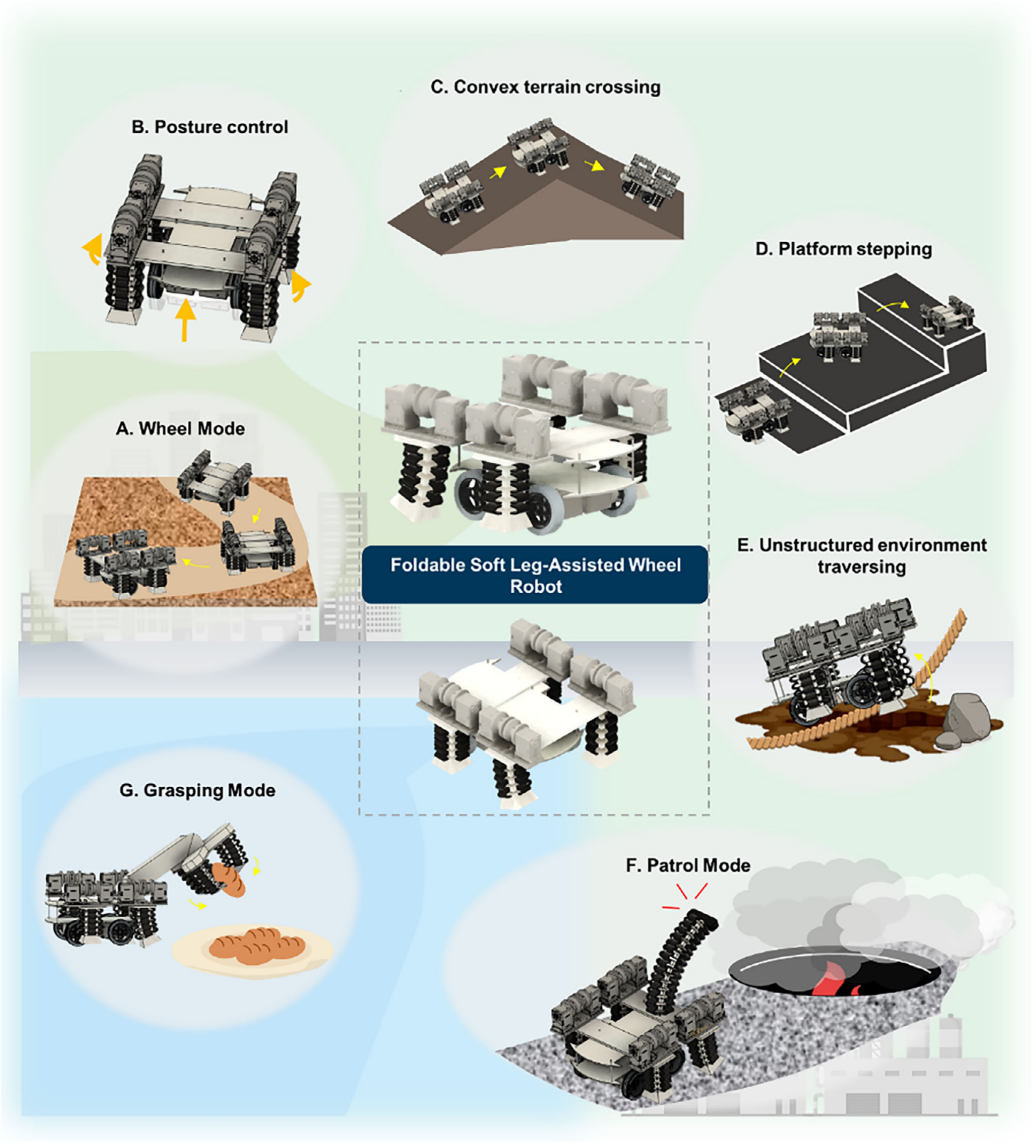
Conceptual illustration of locomotion modes for the FoSLAW. A) Wheel mode for a flat terrain. B) Assist mode for posture control using foldable leg assistants. C) Convex terrain, D) platform, and E) unstructured environment crossing with the assistant‐wheel coupling. F) Patrol mode with the foldable manipulator. G) Grasping mode with the foldable gripper.

In this research, we propose a novel approach to the leg‐wheel robot by integrating foldable soft robotic legs into a wheel robot. Previous studies have developed agile locomotion in mobile robots by arranging multiple soft robotic legs along both sides of the robot body to traverse complex terrain.^[^ ^30^, ^31^, ^32^ ^]^ Motivated by the design paradigm, we built a leg–wheel hybrid robot equipped with four soft robotic legs mounted symmetrically on both sides of the wheel chassis. The Foldable Soft Leg‐Assisted Wheel robot (FoSLAW) demonstrates the multimodal performances across a wide range of realistic mission scenarios (**Figure** **1**). The Foldable Leg Assistant (FoLA) constitutes the key principle of the FoSLAW. When selective cable tension is applied, the robotic legs based on the Foldable Module (FoM) undergo substantial linear deformation and directional bending, which occurs preferentially along a specific direction due to the foldable structure and applied forces. Additionally, the FoLAs exhibit instantaneous mechanical response under external loads due to the dynamics of the foldable structures. Leveraging these features, the FoLAs enable excellent adaptation of the hybrid robot to terrain conditions. For instance, the FoSLAW cruises the flat and compact surfaces by retracting the FoLAs during the wheeled locomotion. In addition, the FoLAs, capable of sustaining the robot weight, are extended to modulate the posture of the robot. The adjustment allows the robot to overcome harsh environments characterized by sudden topographical transitions, thereby significantly enhancing overall maneuverability.

This study also endeavors to contribute to the development of the paradigm in soft robotic legs for leg‐wheel mechanisms in three core aspects. First, a systematic analysis is developed for the foldable soft legs based on modularization. Noting the modular design of the foldable structures, featured by stacking multiple levels to construct a module,^[^ ^42^, ^44^, ^47^ ^]^ step‐by‐step characterization from the foldable module to the robotic leg is conducted, involving simulations and experiments of the mechanical performances. Second, a proof of concept is proposed to evaluate the effectiveness of the foldable soft legs in the leg‐wheel hybrid systems. Mission scenarios that are challenging for the typical wheel robots to traverse are carried out, accompanied by detailed physical interaction techniques of the robot designed to overcome the obstacles. The quantification of the obstacle crossing capabilities is also considered using key parameters that indicate locomotion performances of the hybrid robot. Finally, feasible applications of the FoLA are proposed. End effectors such as manipulators and grippers are designed to augment the mission capabilities of the FoSLAW, leveraging the mechanical characteristics of the modularized foldable structures.

## 2 Results

### 2.1 Foldable Module (FoM)

**Figure 2:**
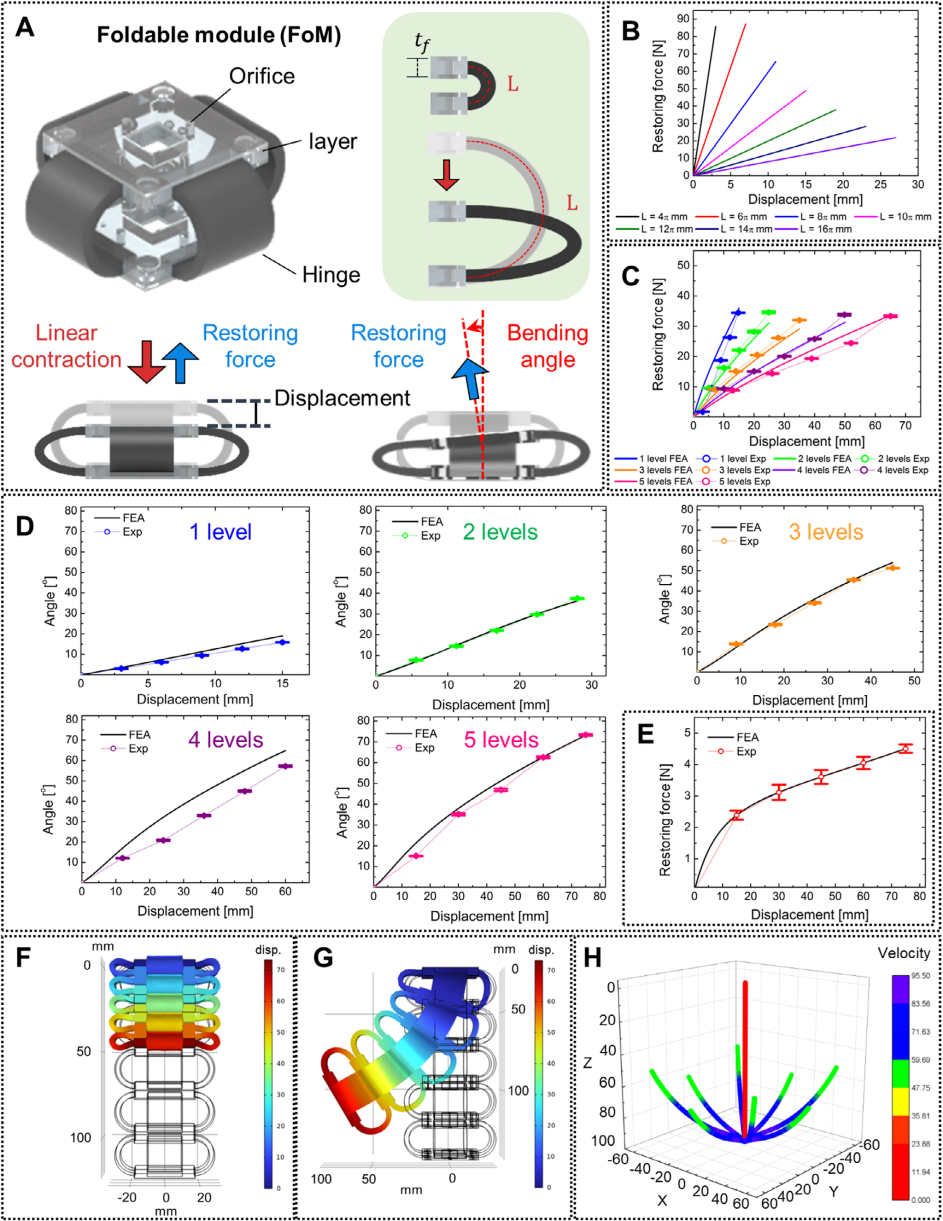
Mechanical characteristics of the FoM. A) The schematic diagram of the FoM and the operating principle. B) FEA simulation results of the restoring force from the FoM with various hinge lengths. C) The restoring force‐displacement curves of the FoMs with various numbers of levels. D) The bending angle‐displacement curves for various levels of the FoMs. E) The restoring force‐displacement curve of 5‐level FoMs on the bending configuration. F) The simulated linear contraction and G) directional bending of 5‐level FoMs. H) Simulated tip trajectories of the FoMs.

The mechanical features of the engineering foldable structure were determined based on its pattern and base material. Previous researches about the soft actuators with bellows tubes have exhibited both large contractile motion and directionality on symmetric configuration.^[^ ^49^, ^50^, ^51^ ^]^ Leveraging the unique features, the conceptual design of the foldable structure was inspired by the bellows tubes (Figure 2A). In particular, the bellows design was simplified leveraging the flexible hinges, which are equivalent to the creases in conventional foldable structure.^[^ ^44^ ^]^ The foldable structure was built from multilevel FoMs, each of which consisted of four hinges and two layers (detailed design and fabrication are introduced in Experimental Section). The key mechanism of the FoM was the restoring force generated when it was contracted. The force can be converted into the driving force of the soft robotic leg, as well as the contraction and release for its deformability. The resilient hinges play a critical role in the mechanism, since the restoring nature of the folded hinges offers the reaction force to the contracted FoM. Since the flexure of the hinge is achieved through anchoring the layers of the FoM,^[^ ^52^ ^]^ the parameter of the hinge length L, which is exposed outside the FoM, can be considered with the thickness of the layer t_*f*_ by $\frac{L}{t_{f}/2}\geq \pi$.

The restoring force analysis of the FoM in response to the contracted displacement was conducted using Finite Element Analysis (FEA) simulation, as shown in Figure 2B. The simulations were performed within solid mechanics interfaces in a stationary study using a commercial software (COMSOL Multiphysics 6.0). The layers and hinges were modeled as a linear elastic material and selected for hexahedral meshing under large deformation (detailed description of the simulation is introduced in Note S1, Supporting Information). Considering the dimension of the cross‐section (20 mm × 3 mm) in prior, the results with the various hinge lengths L were compared. The displacement of the FoM increased continuously as the length L increased, due to the growing distance between the layers of the FoM. However, a disadvantage arose in the form of the reduced restoring force slope, since the hinges became easier to fold when the flexure point moved farther from the anchor points. Considering the tradeoff, the length L was designed to be 10 π to meet the driving force and shape deformability.

The restoring force was derived from the FoM in the overall contraction, which was distributed by the flexure of the individual hinges. The experimental results of the restoring forces from the FoMs with various numbers of levels are presented in Figure 2C. The FoMs were contracted by a force testing machine, and the restoring force on each iteration was measured (detailed description of the experimental setup is available in Experimental Section). The restoring force generated by each FoMs increased up to 35 N as the retraction continued until the contact between the layers in the FoMs was completed. The short error bars indicate that the restoring force results from the FoMs were relatively constant across the structures. The maximum displacement increased up to 65 mm, due to the greater total distance accumulated from the gaps between the layers. The simulation results were also overlapped with the curves. The experimental results show good agreement with the simulation over a wide range of displacements. The observed errors are likely due to differential flexure of the hinges in the FoMs during contraction experiments, whereas the simulation assumes uniformly bent hinges.

Directional bending is the representative mechanical performance of the FoM. When the normal force was applied eccentric to the FoM, the individual folding of the hinges was instantly induced. This resulted in the asymmetric configuration of the FoM, providing the bending angle between the axes of the bottom and top layers. The experimental results of the bending angle with various levels were carried out, as shown in Figure 2D. The zig on the testing machine descended and was in contact with the edge of the FoMs to induce the directional bending (detailed experimental setup is presented in Experimental Section). The results showed a gradual increase in the bending angle as the number of levels increased, accompanied by an increasing range of the displacement of the zig. In particular, a high bending angle of a maximum of 73° was obtained by the FoMs of the five levels, performing a wide range of the zig displacement up to 75 mm. The simulation results were also showcased on each FoM, following well with the bending experiments. The restoring forces in the axis of the bottom level were also generated from the bent FoMs. The difference between experimental and model results was pronounced in 4‐level FoMs. This can be attributed to decreased stiffness of the foldable structure due to local imperfections of the comprising hinges. As shown in Figure 2E, the restoring force from 5‐level FoMs steadily escalated to a maximum of 4.5 N along with the bending. The FoMs maintained their bent configuration, continuously exerting a restoring force until the zig was detached.

The simulation results depicted in Figure 2F,G demonstrate both linear and bending transitions of the FoMs, revealing a wide range of displacements that reflect their deformability. By leveraging that the directional bending was induced by the eccentric normal force, the tip trajectories of the FoMs were simulated based on linear contraction and directional bending caused by the actuation at one or two edges, as shown in Figure 2H. Although a constant value of 21 mm s^−1^ was exhibited by the FoM in linear contraction, a nonlinear behavior of the tip velocity was observed in directional bending. During the 1.1 s motion, the velocity initially soared to 85 mm s^−1^ as the hinges bent freely, and then gradually decreased to 50 mm s^−1^ due to interference caused by the mutual contact between the hinges during bending (Figure S4, Supporting Information). These results indicate that the orientation and mechanical performance of the FoMs can be characterized according to the aspects of the applied inputs.

### 2.2 Foldable Leg Assistant (FoLA)

**Figure 3:**
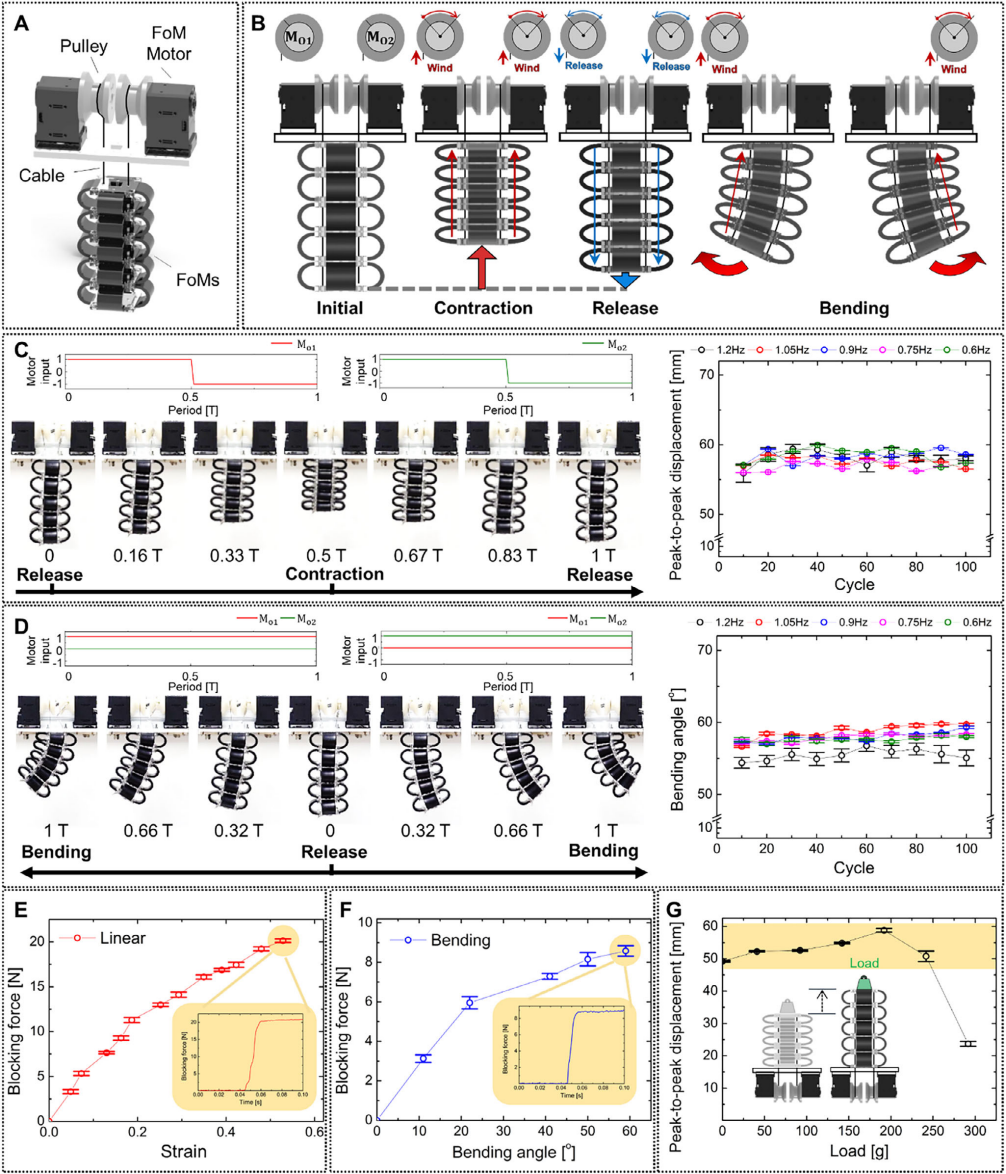
Characteristics of the FoLA. A) Exploded view and key components of the FoLA. B) Schematic of the FoLA mechanism using the FoM motors. Time‐lapse photographs and results of the cyclic tests for the FoLA in C) linear and D) bending actuation with various frequencies. E) Blocking force‐strain curve and F) relation between the blocking force and bending angle of the FoLA. Blocking forces for operating time of 0.1 s are inserted as inset plots. G) Displacement‐payload curve of the FoLA.

The FoLA consisted of the FoMs with five levels and motor‐cable systems, as depicted in Figure 3A (detailed design and configuration of the FoLA are introduced in Experimental Section). Motivated by the unique features of the FoMs, the actuating mechanism of the FoLA primarily relied on cable tension from the FoM motors (Figure 3B). Specifically, linear actuation of the FoLA was achieved through the contraction and deployment of the FoMs, driven by the simultaneous winding and release of the two cables. Not only that, the bending actuation was realized by selectively winding one cable while the other remained inactive.

Cyclic tests were implemented to evaluate both the mechanical performance and operational reliability of the FoLA. Linear actuation was repeatedly conducted with a period of T. The FoLA was contracted as the FoM motors were activated concurrently and returned to its initial state after the activation of the motors at 0.5T (detailed test setup is introduced in Experimental Section). The experimental data reveal that the peak‐to‐peak displacements remain stable over multiple cycles (Figure 3C). Although slight fluctuations were observed, the minor errors measured at every 10 cycles presented the reliability of the FoLA in achieving consistent linear retraction. Moreover, the trend persisted across a range of actuation frequencies, with the average values between 56 and 58 mm from 0.6 to 1.2 Hz, affirming the robustness of the FoLA (detailed results are introduced in Table S2, Supporting Information). Furthermore, as shown in Figure 3D, the bidirectional bending was also performed by alternating the motor activation (detailed characterization method is introduced in Experimental Section). In the results, the measured average bending angles showed a relatively steady pattern over the cycles, maintaining their consistency between 55° and 58°. Similar results were also presented under varying frequencies, further validating the reliable bending performances of the FoLA (detailed results are introduced in Table S2, Supporting Information).

The blocking forces of the FoLA during the linear actuation were also measured in Figure 3E. Linear strain was calculated as the ratio of the displacement to the initial length of the FoMs (123 mm) attached to the FoLA (detailed test setup is showcased in Experimental Section). The results exhibited a similar trend to that of the FoMs, showing an increasing curve across the strain range with small errors. Herein, a rapid increase in blocking force was observed immediately, as well as activation of the FoLA within ≈20 ms, and the force was maintained thereafter. The soaring in the blocking force was also identified in the bent actuation of the FoLA, as depicted in Figure 3F. The blocking forces progressively increased and reached their peaks as the bending angle approached maximum of 66°. The FoLA actuation experiment is available in Movie S1 (Supporting Information).

In terms of blocking force and bending angle performances, the results suggested that the FoLA held promise as a dexterous robotic leg, exhibiting robustness to external loads in both linear and bending configurations, as well as reliability under repeated activation. Figure 3G shows the peak‐to‐peak displacement with the payload, which was evaluated by placing a weight on the FoLA. The system achieved noticeable displacement under loads up to 250 g, which was ≈125% of the weight of the FoMs. However, a sharp decline in displacement was observed when the weight exceeded 300 g, at which point the displacement was reduced to approximately half of the lift range. The reduction was perhaps attributed to the elastic buckling,^[^ ^53^ ^]^ caused by the combined effect of the self‐weight of the FoMs and the applied weight.

### 2.3 Foldable Soft Leg‐Assisted Wheel Robot (FoSLAW)

**Figure 4:**
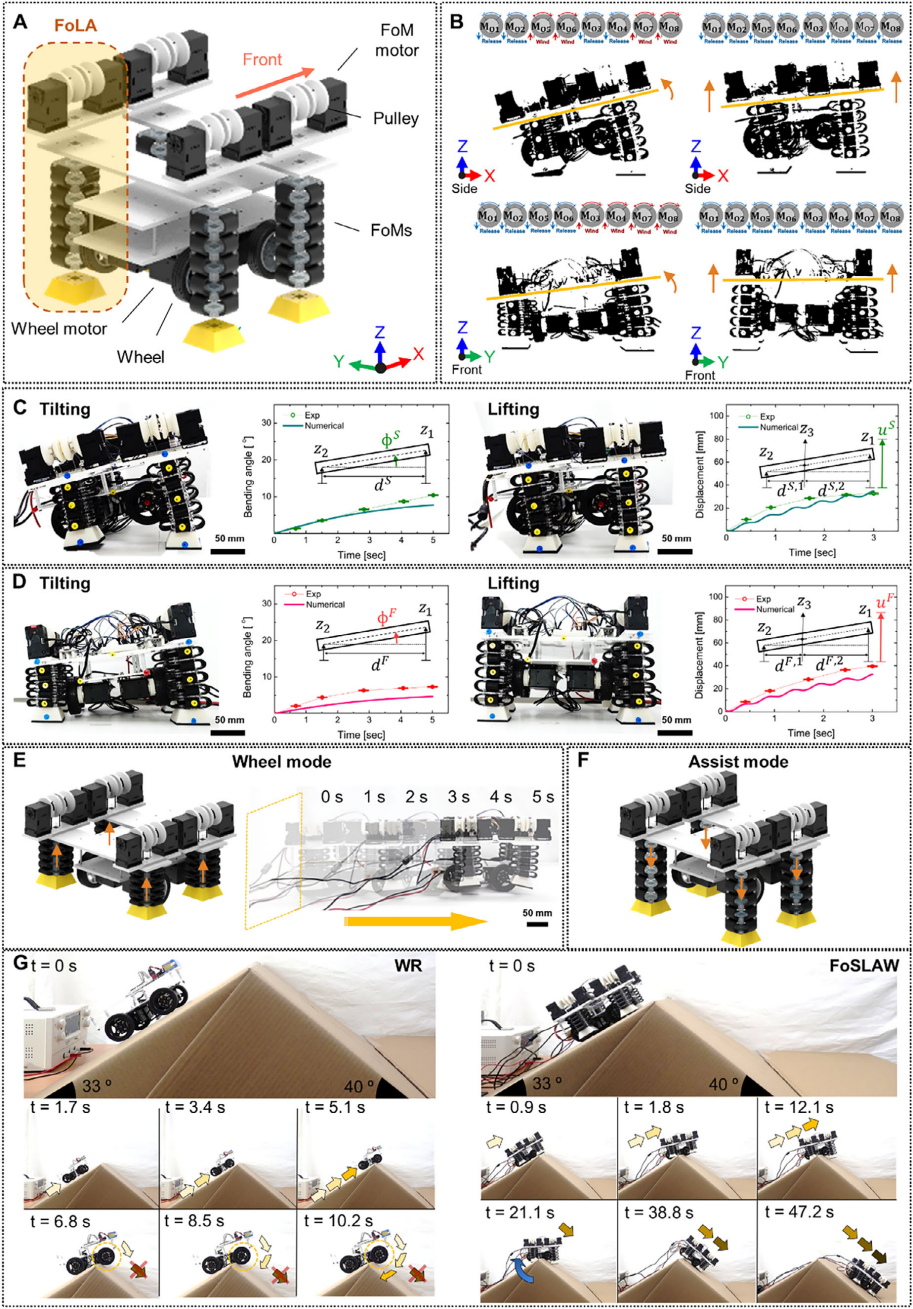
Design and operation of the FoSLAW. A) Structural design of the FoSLAW. B) Posture adjustment with the motor diagram of the FoSLAW. The real time performance photographs and comparison results between experimental and numerical models of the FoSLAW at the C) side view and D) front view. Schematic of E) wheel mode with the retracted FoLAs for high speed locomotion and F) assist mode using the expansion of the FoLAs. G) Time lapse image sequence of tilting and lifting maneuver to traverse a convex terrain.

Figure 4A shows the conceptual design of the FoSLAW with its components. Specifically, multiple FoLAs were employed on the sides of the four wheels by placing the motor‐cable systems over the robot body (detailed structural design of the FoSLAW is available in Experimental Section). The FoLAs were controlled individually by activating the eight FoM motors in the FoSLAW. Therefore, the tilting motion of the robot was exhibited by deploying the front FoLAs, which was caused by releasing the four cables, while retracting the rear FoLAs (Figure 4B). The inclination was also conducted by deploying the two FoLAs on the left of the robot and retracting the right two FoLAs. Furthermore, the robot was lifted immediately by releasing all cables equipped to the FoLAs.

The postures of the FoSLAW in motion were captured and presented in Figure 4C,D. By extending only the front FoLAs, the front wheels were completely floated, making the robot tilt backward (detailed control method is introduced in Experimental Section). Additional tilting was also performed by the FoSLAW by deploying the FoLAs on the side, allowing the floating of the side wheels. The experimental results show that the tilting angle increases as the FoLAs were gradually extended. The curves reached the maximum values until the expansion of the FoLAs ceased due to the restoring force balancing the weight of the FoSLAW. Complete floating of the wheels on the robot was exhibited in the lifting motion by fully deploying all the FoLAs. The displacements of the FoSLAW in the lifting also presented increasing trends, showing the maximum distance that is ≈23% of the robot body height. The predictions based on the mathematical modeling were covered on the graphs to verify the experiments. In the modeling, the FoMs within the FoLAs were simplified into a dynamic model, while the cable tensions generated by the FoM motors were treated as external loads on the FoLAs as

(1)

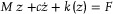

where *M*, *c*, *k*(z), and *F* represent the mass element, damper element, spring element, and external load to the FoLA, respectively. Therefore, the FoSLAW can be modeled using a matrix form of the equations of motion as (detailed dynamic model is introduced in Note S2, Supporting Information)

(2) $$\begin{array}{cc} & \left[\begin{array}{ccc} m_{1} & 0 & 0\\ 0 & m_{2} & 0\\ 0 & 0 & m_{3} \end{array}\right]\left[\begin{array}{c} {\ddot{z}}_{1}\\ {\ddot{z}}_{2}\\ {\ddot{z}}_{3} \end{array}\right]+\left[\begin{array}{ccc} c_{1}+c_{3} & 0 & -c_{1}\\ 0 & c_{2}+c_{4} & -c_{2}\\ -c_{1} & -c_{2} & c_{1}+c_{2} \end{array}\right]\left[\begin{array}{c} {\dot{z}}_{1}\\ {\dot{z}}_{2}\\ {\dot{z}}_{3} \end{array}\right]+\left[\begin{array}{ccc} 2k_{1} & 0 & -k_{1}\\ 0 & 2k_{1} & -k_{1}\\ -k_{1} & -k_{1} & 2k_{1} \end{array}\right]\left[\begin{array}{c} z_{1}\\ z_{2}\\ z_{3} \end{array}\right]+\\ & \;\left[\begin{array}{c} k_{2}{\left(z_{1}-z_{3}\right)}^{2}+k_{3}{\left(z_{1}-z_{3}\right)}^{3}+k_{2}z_{1}^{2}+k_{3}z_{1}^{3}\\ k_{2}{\left(z_{2}-z_{3}\right)}^{2}+k_{3}{\left(z_{2}-z_{3}\right)}^{3}+k_{2}z_{2}^{2}+k_{3}z_{2}^{3}\\ k_{2}{\left(z_{3}-z_{1}\right)}^{2}+k_{3}{\left(z_{3}-z_{1}\right)}^{3}+k_{2}{\left(z_{3}-z_{2}\right)}^{2}+k_{3}{\left(z_{3}-z_{2}\right)}^{3} \end{array}\right]=\left[\begin{array}{c} F_{1,i}^{j}\\ F_{2,i}^{j}\\ 0 \end{array}\right] \end{array}$$

The experimental results showed a trend similar to the predictions. The discrepancy appears to be caused by the vibration induced by the FoM motors during the activation.

The dynamic motion of the FoLAs can endow multimodality to the FoSLAW. The robot was capable of traversing flat and smooth surfaces in the wheel mode while fully retracting the FoLAs not to interfere with the locomotion (Figure 4E). Wheel mode was evaluated to quantify the effect of the additional weight introduced by the FoLAs. The FoSLAW (3 kg) cruised on a flat surface, measuring the energy consumption of the wheel motors. Also, the experiments were conducted by the wheel robot (1.2 kg), and repeated five times by attaching the additional weight on the main plate. Energy efficiency *n_w_* of the wheel mode can be given by (detailed calculation of the *n_w_* is available in Note S3, Supporting Information)

(3) $$n_{w}=\frac{mgd^{w}}{W}$$

where *m* is the mass of the mobile robot, *g* is gravitational acceleration, *d^w^* is the cruise distance, and *W* is the total energy consumption. The wheel robot exhibited a nearly constant efficiency of ≈0.20 as its weight increased to 3.2 kg, which was ≈250% of the weight of the wheel robot. The FoSLAW also achieved an efficiency of 0.21, indicating that the additional weight from the soft appendages had only a minor influence on the efficiency of the robot in wheel mode (Figure S10, Supporting Information). In addition, by deploying the FoLAs, the robot can adjust its body posture, such as inclination angle and orientation (Figure 4F). Figure 4G demonstrated an interesting maneuver of the FoSLAW in a harsh environment, leveraging the multimodality. The wheel robot became immobilized at the end of the ramp while attempting to traverse a convex terrain. This was because the robot contacted the apex of the break over angle, which prevented the robot from mobilizing further in its direction. The break over angle of the wheel robot can be approximately calculated as^[^ ^54^ ^]^

(4) $$\mathrm{Break}\;\mathrm{over}\;\mathrm{angle}\;=2\;\tan^{-1}\left(\frac{2b}{a}\right)$$

where b = 16.5 mm is the ground clearance and a = 120 mm is the wheelbase. The break over angle was 30.7°, which was less than the convex terrain angle of 73°. By deploying the FoLAs, the robot incrementally lifted and recovered driving power by contacting the front wheels to the ground. The experiment implied that the FoLAs can provide excellent obstacle crossing capability beyond the design limitations of the wheel robot. The terrain traversing sequence is displayed in Movie S2 (Supporting Information).

**Figure 5:**
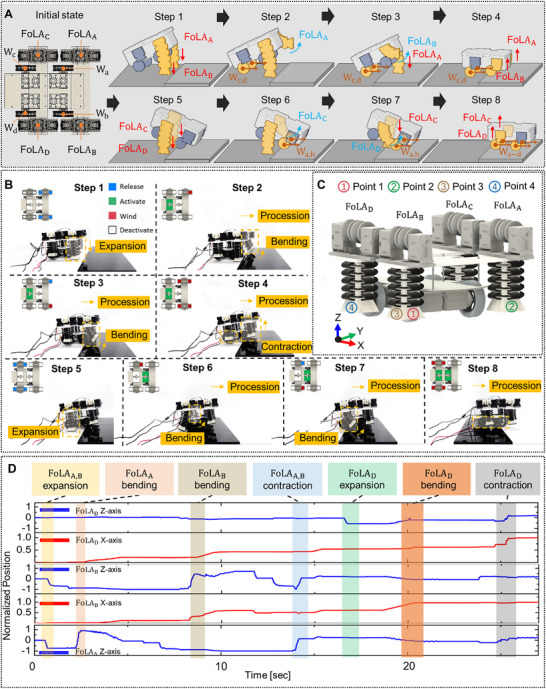
Platform stepping of the FoSLAW. A) Schematic description and B) snapshots of the stepping scenario. C) Setup of stepping locomotion tracking. D) Real‐time normalized position data of the FoSLAW stepping along various axes.

Leveraging this capability, a hybrid locomotion mechanism was developed that enabled the independent motor controls of the FoSLAW to adapt to the environmental conditions in certain mission scenarios. The platform stepping mechanism of the FoSLAW is illustrated as one of the mission scenarios in Figure 5A. In the first phase (Steps 1 to 4), the robot securely placed its two front wheels onto the platform, positioning the rear wheels adjacent to the platform. The two front FoLAs alternately performed retraction and deployment to either support the body weight or step onto the platform. In the second phase (Steps 5 to 8), the robot elevated its rear wheels onto the platform by activating the two hind FoLAs in coordination with the driving of the front wheels, and ultimately completed the ascent by fully mounting the platform (detailed explanation for the stepping scenario is introduced in Note S4, Supporting Information). The maneuver was executed through individual control of the motors (Figure 5B). The expansion of the initially retracted FoLA was achieved by releasing the cables of the two FoM motors constituting the FoLA, whereas the contraction was performed by simultaneously winding both cables. Notably, directional bending of the FoLA was accomplished through selective actuation, in which only one cable was wound. Subsequently, rotation and progress of the robot were conducted through the driving of the wheel motors (detailed motor control sequence is depicted in Figure S11, Supporting Information). The experimental results of the tilting angle by the contraction and expansion of the FoLAs and their predictions were also covered in Figure S12 (Supporting Information). The variations in terrain can be incorporated into the dynamics model through coordinate transformation and by accounting for initial conditions.^[^ ^55^ ^]^ The numerical outputs exhibit a tendency to follow the experimental results, showing minor errors within 4% (detailed dynamic model is introduced in Note S2, Supporting Information). Consequently, the mobile robot successfully overcame the 10 mm‐high platform. The platform stepping experiment is displayed in Movie S3 (Supporting Information). A 90% success rate and an average traversal time of 31.1 s were exhibited by the FoSLAW under 50 trials (detailed performance results and metrics are available in Figure S13 and Table S4, Supporting Information). Here, the result indicates that the FoSLAW failed to step up the platform when the initial displacement between the FoSLAW and platform exceeded 190 mm, which is ≈160% of the average displacement. This may be because FoSLAW moved away from its intended location and did not engage with the platform while approaching the platform from a larger distance.

To quantitatively validate the platform stepping scenario, the actuation sequences of the FoLAs were evaluated using the tracking software. Cameras were positioned to capture both front and side views, and the tracking points were attached to the front and side FoLAs for movement analysis (Figure 5C). The relative positions obtained from the tracking data are presented in Figure 5D. Initially, the FoLAs were fully retracted, and the wheels remained stationary. The measured displacements along the z‐ and x‐axes were normalized with respect to the maximum extension of the FoLAs (30 mm) and total travel distance of the hybrid robot (245 mm), respectively. The positions in the *z*‐axis decreased, reflecting the downward motion of the FoLAs in expansion. Conversely, bending of the FoLAs led to an increase in the *z*‐axis position, as the tracked shoes rose upward. In particular, when the robot went up the platform, the *z*‐axis values in deployment remained elevated above zero, due to the platform height. Meanwhile, the *x*‐axis position gradually increased as the mobile robot progressed forward. Furthermore, Cost of Transport (CoT) was calculated to quantitatively evaluate the platform stepping using the equation as^[^ ^56^ ^]^

(5) $$\mathrm{CoT}\;=\frac{W}{mgd}$$

where *d* represents total locomotion distance of the robot. Effective energy efficiency η_*p*_ of the FoSLAW can also be achieved using the equation as

(6) $$\eta_{p}=\frac{mgd^{P}}{W^{P}}$$

where *d^P^* and *W^P^* represent the displacement of the FoSLAW and particular energy consumption of the motors during the sequences from FoLA_A,B_ expansion to FoLA_D_ contraction (detailed modeling and calculation of the CoT and η_*p*_ are available in Note S3, Supporting Information). The CoT and η_*p*_ result for platform stepping were 10.82 and 0.18, respectively.

**Figure 6:**
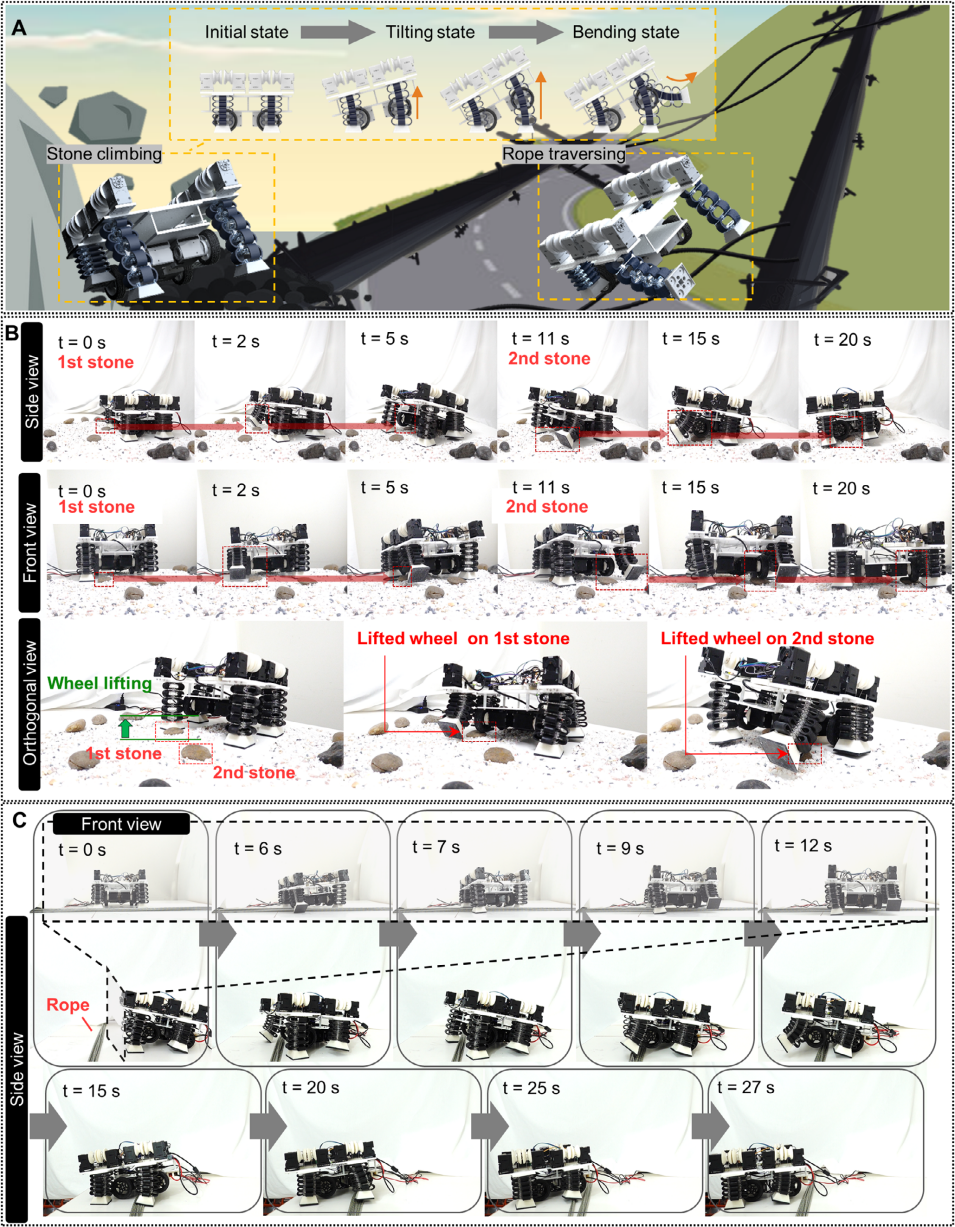
Obstacle traversing of the FoSLAW. A) Schematic description of unstructured scenarios. Snapshots of the FoSLAW operation on B) gravel terrain and C) flexible rope.

The hybrid locomotion mechanism, which integrates the FoLAs and wheels, is also capable of overcoming unstructured environments (Figure 6A). A locomotion technique was developed to overcome the obstacles by actively placing the wheels on top of them. In the method, the front FoLAs were deployed to lift the wheel upward, allowing them to make contact with the obstacles using the bottom parts of the wheel. The locomotion for climbing and overcoming a series of stones, one of the mission scenarios, was observed from the side, front, and orthogonal views and presented as a time‐lapse in Figure 6B. First, the front right FoLA was bent, and the rear wheels were driven to rotate the robot body so that the front wheel moved over the first stone on the left. After that, the front FoLA was contracted so that the front wheel made contact with the stone. Then, the robot traversed the stone by driving the front wheel. Similar locomotion was also implemented on the second stone. A unique feature of the technique was that the wheels were lifted by the FoLAs so that their bottom surface could engage with the unstructured stones. This allowed the robot to climb over the stones, enabling novel obstacle negotiation that was distinct from the technique using the wheel power to cross over the obstacles. The stone traversing experiment is captured in Movie S4 (Supporting Information)

The other representative example of uneven obstacles was a flexible rope. When a typical wheel robot attempts to traverse the rope, the rope can become entangled around the wheels. This phenomenon may increase resistance against wheel rotation, potentially leading wheel systems to be completely immobilized. The rope traversing of the robot using the locomotion technique was demonstrated in Figure 6C. The front right FoLA crossed the rope by retraction and deployment, raising the front right wheel onto the rope. Then, the other front FoLA repeated the actuation so that the front two wheels were completely stepped over the rope. After contracting the FoLAs, the robot crossed over the rope by mobilizing the wheels. The rear FoLAs and wheels also crossed the rope with the same process. The locomotion by activating the FoLAs that avoided the rope decreased the wheel surface in contact with the flexible rope during the traversing process, which may reduce the possibility of the rope entanglement around the wheels. The rope traversing experiment is displayed in Movie S5 (Supporting Information). The experiments of traversing flexible rope and gravel terrain were repeated for 20 trials showing 85% and 90% success rates, respectively (detailed performance results and metrics are available in Figure S14 and Table S4, Supporting Information). Herein, the FoSLAW failed the unstructured environment scenarios when the tilting angles of the FoSLAW in contact with the gravel terrain and flexible rope were less than ≈35% of the average value. The results suggest that such low tilting angles may provide insufficient front wheel lifting to climb over or avoid the obstacles. Table S6 (Supporting Information) summarizes the FoSLAW and representative robot groups, including leg‐wheel hybrid, retractable leg, and soft foldable robots in terms of body height, weight, obstacle type, mechanism, and locomotion type. The leg‐wheel robots typically exhibit larger body dimensions and heavier weights.^[^ ^14^, ^15^, ^16^ ^]^ The robots demonstrated traversal experiments of simple terrain, such as a flat surface and a single obstacle, showing leg‐wheel switching mechanisms. The robots with retractable legs usually incorporate transformable wheel mechanisms with adjustable wheel locomotion, covering relatively small body sizes and low weights.^[^ ^17^, ^18^, ^19^ ^]^ In contrast, soft foldable robots represent a miniaturized and lightweight category, featuring foldable structures that enable morphing and crawling locomotion, effective in unstructured environments such as low walls, stairs, sand, and narrow channels.^[^ ^39^, ^40^, ^41^ ^]^ The proposed FoSLAW, whose design was motivated by the existing robots, exhibited a moderate body size and weight, demonstrating a wheel and soft‐leg hybrid mechanism using synergistic leg‐wheel locomotion to adjust and traverse complex terrain, including flat surface, convex terrain, flexible rope, and gravel terrain.

### 2.4 Applications for FoM

**Figure 7:**
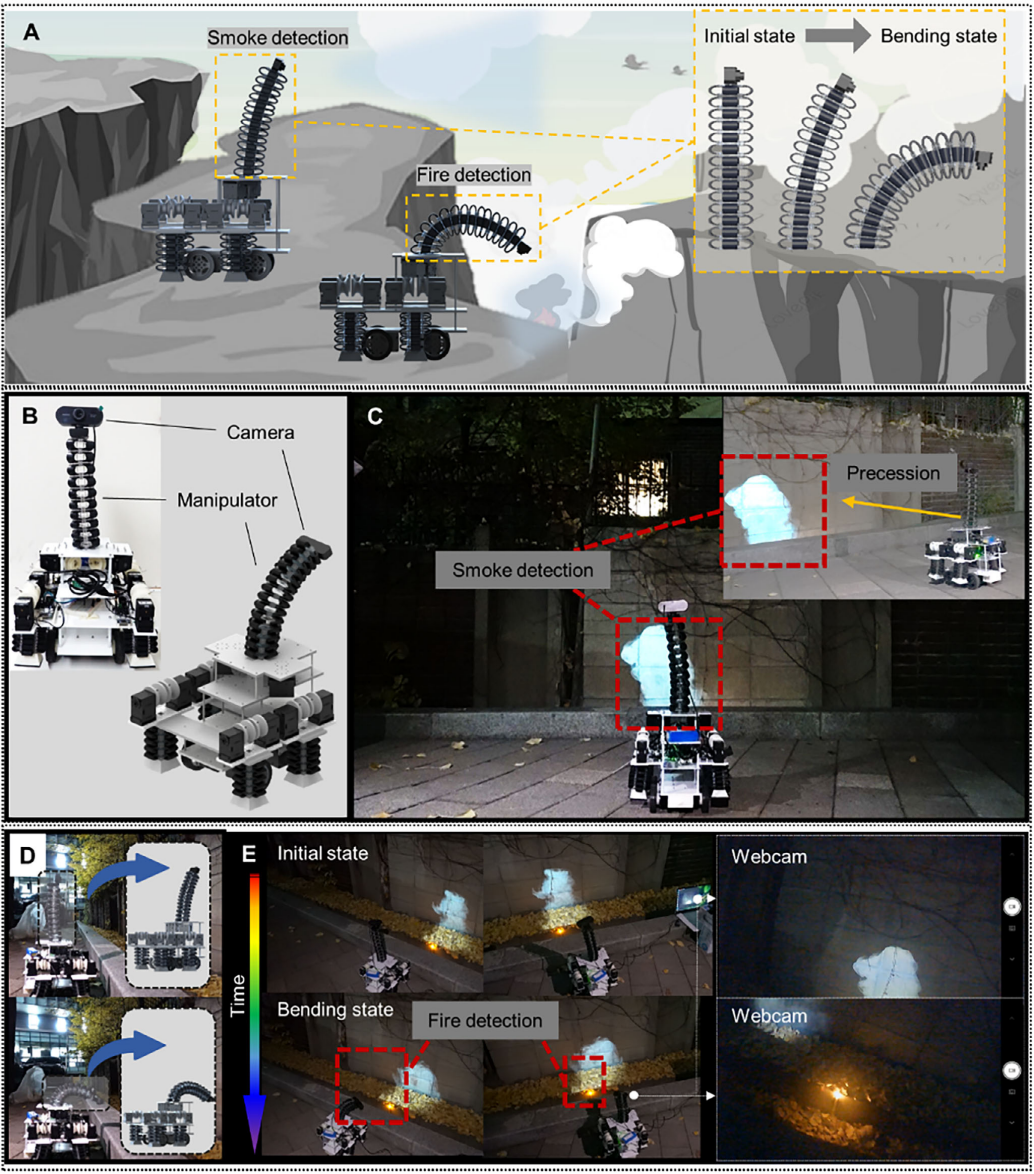
Patrol mode of the FoSLAW with a foldable manipulator. A) Schematic description of smoke monitoring. B) Structural design of the manipulator installation on the FoSLAW. Snapshots of the patrol scenario: C) smoke detection and procession, D) manipulator bending using the motors, and E) fire source detection and real‐time monitoring.

Although the hybrid locomotion and its technique were demonstrated through accomplishing the mission scenarios in various environments, the robot can struggle to physically approach certain facilities with relative steps, such as pits, drains, and underground places (Figure 7A). A foldable manipulator that can approach the environments can be designed leveraging the deformability and stacking configuration of the FoMs. In particular, FoMs with 15 levels were utilized to construct the manipulator. The additional motors were attached to the motor panel so that two cables penetrated through two adjacent orifices of the FoMs. After installing a camera on top, the manipulator was mounted to the FoSLAW (Figure 7B) (detailed description and dimensions of the manipulator‐equipped FoSLAW are showcased in Figure S15A,B, Supporting Information). The robot first captured an image of smoke in a drain, which is partially blocked by curbstones, using the camera on the tall manipulator, and proceeded to the spot (Figure 7C). Afterward, large directional bending of the manipulator was conducted by winding the cables (Figure 7D). Consequently, the robot successfully observed and monitored the source of the fire model in the drain below the ground (Figure 7E). Therefore, the FoSLAW conducted the patrol mode by enhancing its functionality by equipping the foldable manipulator, which was capable of approaching and monitoring the specific facilities. The FoM manipulator activation of the FoSLAW is available in Movie S6 (Supporting Information).

**Figure 8:**
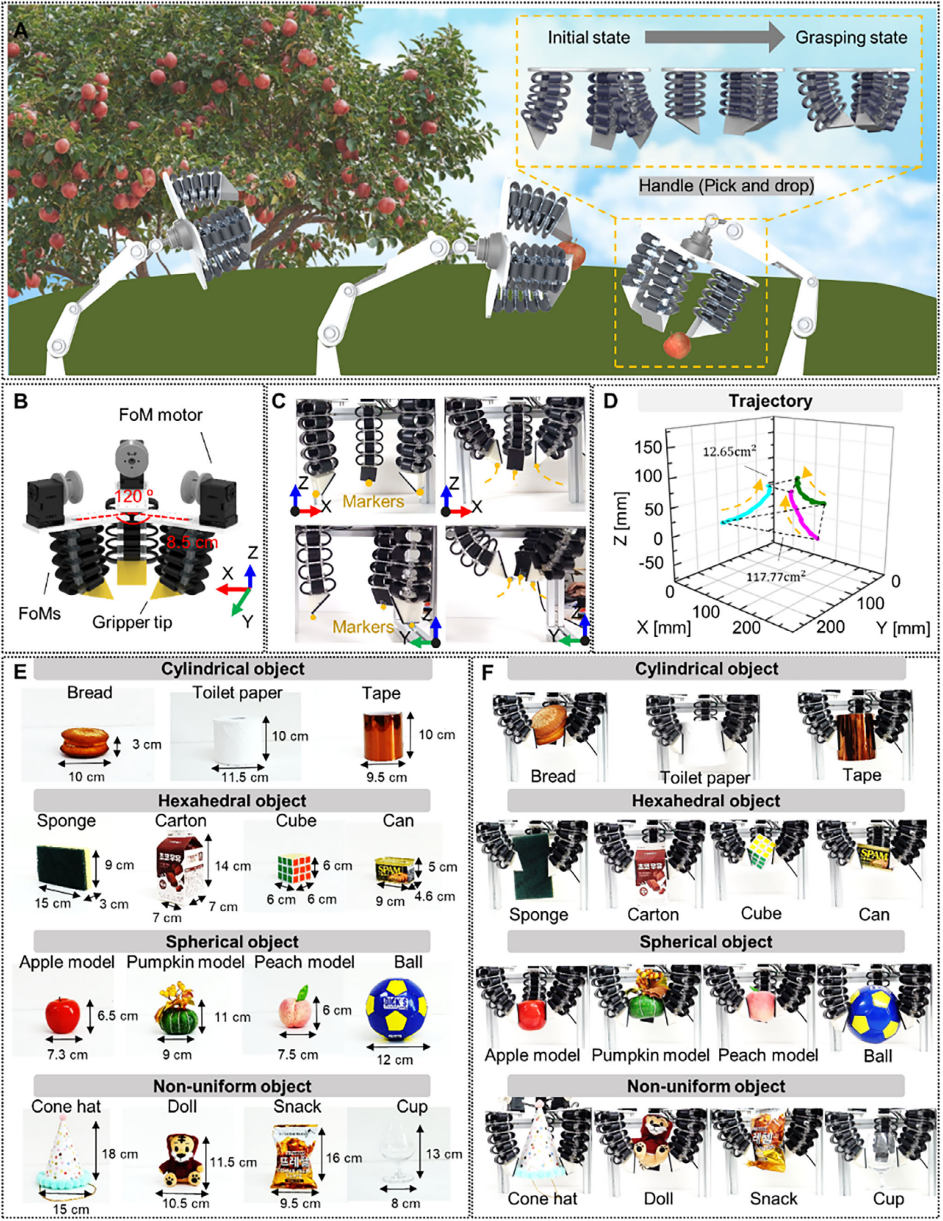
Grasping application of the FoM. A) Schematic description of the grasping. B) Design of the foldable gripper. C) Setup of gripper tracking. D) Trajectory of the foldable gripper. E) Arrangement and F) photographs of test objects: bread (97.5 g), toilet paper (128.5 g), tape (266.0 g), sponge (18.3 g), carton (24.5 g), cube (64.2 g), can (220.1 g), apple model (11.4 g), pumpkin model (28.7 g), peach model (85.4 g), ball (186.8 g), cone hat (17.5 g), doll (59.9 g), snack (91.0 g), and cup (165.4 g).

The blocking force performance of the FoLA on bending configuration also suggests the potential of a dexterous foldable gripper as an application. Directional bending can be induced by the FoLAs facing each other so that their tips are assembled, which can grip soft and fragile objects (Figure 8A). Each foldable finger was designed by combining 5‐level FoMs and single motor‐cable system, and attaching a gripper tip to the end of the FoMs (Figure S16A, Supporting Information). The tension of the cable caused the finger to be bent in a specific direction, thereby implementing a gripping motion in which the tip contacted and supported the objects (Figure S16B, Supporting Information). Three gripper fingers were arranged facing each other with the same dihedral angle of 120°, having a distance of 8.5 cm from the gripper axis (Figure 8B). The tip trajectories of the gripper were verified through activation experiments (Figure 8C). As the fingers were bent, the gripper tips were gathered to the gripper axis. Real‐time tracking was conducted focusing on the markers at the edge of the tips, and their coordinates were evaluated. Figure 8D shows the experimental results with the positions of the tips. The gripper tips initially presented a triangular shape with an area of 117.77 cm^2^. The area decreased as the bending continued and ended up reaching to 12.65 cm^2^. The results indicated that the device was capable of gripping objects within a wide range of workspace within the areas. A series of experiments was also conducted, grasping objects with various shapes and weights. The objects were classified into cylinders, hexahedrons, circles, and irregular shapes (Figure 8E). The gripper was contacted and firmly grabbed the objects by operating the FoM motor. The objects were stably grabbed and held even though they had various sizes and weights (Figure 8F). In particular, the foldable fingers on the gripper were adapted to the surface of the objects by deforming their shape. Eventually, the gripper can hold the objects with simple motor control. The results suggest that the FoM had the potential to be applied as a universal gripper. The object grasping experiment is displayed in Movie S7 (Supporting Information).

## 3 Conclusion

In this study, we provided new insights into robotic leg designs for leg‐wheel robots. Noticing the variable stiffness and deformability of the foldable structures, we focused on the soft leg assistant named FoLA, whose design was inspired by the FoMs that were capable of generating driving force and large transitions. The bottom‐up design approaches from the FoM to the FoLA were proposed using a detailed process. We also developed a novel hybrid locomotion mechanism combining the soft robotic legs with the rigid wheels to traverse various obstacles.

We detailed the unexplored structural design and mechanism through a series of experiments. Characterization was presented gradually from the FoMs to the FoLA, evaluating the mechanical performances with various design parameters and conditions. The obstacle crossing capabilities of the FoSLAW, leveraging a hybrid locomotion mechanism, were also showcased explaining locomotion techniques for the mission scenarios such as the slope, platform, stone, and flexible rope.

In experiments, we showed that the foldable robotic legs can be utilized to provide obstacle traversing capabilities to the leg‐wheel robot. Blocking force performance showcased by the FoLA was enough to permit overall detachment of the rigid wheels from the ground. The large deformations of the FoLA in both linear and bending directions allowed the wheels to selectively contact uneven environments, enabling new locomotion techniques such as stepping and climbing that were not feasible for the conventional wheel robots requiring continuous ground contact. Additionally, the foldable manipulator and gripper were proposed as feasible applications of the FoM to augment the functionality of the proposed structure. We believe our research can contribute to unlocking dexterous and versatile soft robotic systems capable of enhancing the functionalities of the leg‐wheel robots.

Since open‐loop control was utilized for the mobile robot, differences in the conditions of the mobile robot interacting with the complex environments were observed, which affect the success rate in the obstacle traversal experiments. Therefore, further research will focus on enhancing the intelligence of the mobile robot. In future developments, IMU sensors will be integrated into the foldable mechanisms to enable real‐time activation sensing and continuous data transmission to the main processor, thereby facilitating closed‐loop control.^[^ ^55^, ^57^ ^]^ Furthermore, active control of cable‐driven systems using mechanical intelligence will also be taken into account for improving the dexterity of the foldable assistants.

## 4 Experimental Section

### Structural Design of FoMs

The foldable structure consisted of the multiple levels of the FoMs that were stacked vertically. As shown in Figure S1A (Supporting Information), the FoM consisted of two layers and four hinges. Each layer consisted of a layer core (35 mm × 35 mm × 1 mm) and two layer covers (35 mm × 35 mm × 2 mm). Four holes were located at the corners for the bolt connections, whereas four orifices were near the axis for cable penetration. Edges of the layer core were notched (20 mm × 4 mm) to allow clearance for the hinges. The cavity (10 mm × 10 mm) in the middle of the layer was for the lightness of the FoM. Four hinges were prepared for the FoM to be equivalent to the creases of conventional foldable structures (Figure S1B, Supporting Information). Each hinge was in a hexahedral shape with a cross‐section (20 mm × 3 mm). The length (L + 8 mm) can be determined for tuning the mechanical performances of the FoMs.

### Fabrication Process of FoMs

The fabrication process of the FoMs is depicted in Figure S2 (Supporting Information). The acrylic board was cut by a laser machine (FUSION EDGE 12, EpilogLaser) with computer‐aided design (CAD) (Fusion360, Autodesk). A fire extinguishing system was used to prevent additional cracks on the cutting sections from residual heat. The styrene‐butadiene rubbers (SBR rubber, Jingmei) were cut by a straw cutter and bonded on the notches of the layer cores by firmly gluing the contact surfaces. At last, the layer cores were stacked each other in the axial direction with the layer covers and interlocked to the series of bolts and nuts in the holes.

### Experimental Setup of the FoMs

The restoring forces from the FoMs were obtained by measuring the reaction force of the structures on contraction. The FoMs were aligned and attached to a force testing machine (MCT‐2150, A&D co., Ltd), as shown in Figure S3A (Supporting Information). The S‐beam sensor was in contact with the top layer of the FoMs and calibrated to zero. The FoMs were contracted as the sensor descended gradually at a constant speed of 200 mm min^−1^ until the adjacent layers were completely in contact. The axial displacements and reaction forces from the FoMs were obtained and recorded simultaneously, as depicted in Figure S3B (Supporting Information). Then, the directional bending of the FoMs was also evaluated. A L‐shaped zig was attached to the S‐beam sensor and compressed the edge of the FoMs at a constant speed. The bending angles were obtained using the relative coordinate of the tracking points on the vertices through a digital camera (DSC‐RX100M7, Sony) at a rate of 60 frames s^−1^ and post‐processed by commercial software (ProAnalyst Motion Analysis Software, Xcitex). The restoring forces from the FoMs in bending deformation were also measured, as well as the displacement of the zig, as shown in Figure S3C (Supporting Information). Each type of experiment was conducted with 10 repeats, and the average results were calculated.

### Structural Design of the FoLA

The FoLA, which was influenced by a cable‐driven foldable actuator, was mainly organized with one 5‐level FoMs and two motor‐cable systems. To construct the system, a servo motor (XC430, Robotis) was connected with a 3D‐printed acrylonitrile butadiene styrene (ABS) pulley to which a titanium cable was wound. The motors and FoMs were assembled on the FoLA plate made of an acrylic board. The cables penetrated the FoMs and were tied to the bottom layer (detailed dimensions of the components are available in Figure S5A, Supporting Information).

### Characterization Method of the FoLA

The cyclic tests were conducted by the FoLA at various control frequencies. First, the axial displacement of the FoLA was investigated. The cables were wound simultaneously, and the FoMs was contracted, as shown in Figure S5B (Supporting Information). Then, the FoMs returned to the initial length as the cables were released. The tracking point on the bottom of the FoMs was recorded for 100 cycles and post‐processed to evaluate the peak‐to‐peak displacement. Then, the cyclic bending was also performed, as depicted in Figure S5C (Supporting Information). The directional bending was conducted by winding one cable while deactivating the other cable. The tracking points on the vertices of the bottom were filmed, and their relative coordinates were also processed to calculate the bending angle. The motor speed was adjusted based on the control frequencies between 0.6 and 1.2 Hz. Subsequently, the blocking force of the FoLA was measured on the axial and bending deformation. As shown in Figure S5D (Supporting Information), in the case of the axial deformation, a force sensor (RFT80‐6A02, Robotous) was in contact with the FoLA in linear contraction and adjusted to zero. The blocking force was generated as the FoLA was extended by releasing the cables and blocked by the sensor, and was obtained using Python 3.8 software on a PC. The blocking force was also evaluated on the bending deformation. The sensor was calibrated to zero in contact with the FoLA in directional bending and gauged the reaction force from the FoLA as the cable was released. Each test was conducted with 10 repeats, and the results were averaged.

### Structural Design of the FoSLAW

The Wheel Robot (WR) consisted of one body and four wheels. The two main acrylic plates (252 mm × 110 mm × 5 mm) of the body were in parallel and assembled with six support bars. Each wheel was equipped with an independent motor, achieving four‐wheel drive for the robot (detailed dimensions of the WR are available in Figure S6, Supporting Information). The FoSLAW refers to the assembly of the WR and four FoLAs. The FoLAs were placed on the top of the WR body, facing the FoMs downward. A 3D‐printed ABS shoe (50 mm × 50 mm × 20 mm) was attached to each foldable structure in order to compensate for the reduced height of the FoLAs due to the robot weight and to increase friction with the contact surface. The connecting plates, which were perpendicular to the WR, were coupled to the FoLAs so that the FoMs could be separated from the wheels. The arrangement prevented the FoLA activation from interfering with the wheel drive (detailed dimensions of the FoSLAW are available in Figure S7, Supporting Information).

### Control Method of the FoSLAW

Figure S8 (Supporting Information) illustrates the motor control diagram of the FoSLAW. The wheel motors **M**_**W**1 − 4_ (XH540, Robotis) were connected to a main controller (OpenCR, Robotis) and powered by a 12 V supply. The control board received velocity commands transferred from a PC through a universal asynchronous receiver/transmitter (UART). Individual wheel drives can be conducted using separate and sequential commands to the wheel motors. The FoM motors **M**_**F**1 − 8_ were connected to four embedded controllers (OpenRB, Robotis) and powered by a DC power supply (TDP‐3010B, TOYOTECH). The motor position commands from the PC were transmitted to the controllers through inter‐integrated circuit (IIC) ports on the main controller. The commands allowed the FoSLAW to be switched between the drive mode, in which fast locomotion of the FoSLAW by wheel drive can be conducted with the contracted FoLAs, and the assist mode, in which posture control of the FoSLAW can be performed by coupled activation of the FoLAs and wheel motors.

### Experimental Setup for Tracking the FoSLAW

The tracking points were attached to the tip of the FoMs in the FoLA. Herein, four points were used to be tracked in both the side and front views. The displacement of the FoSLAW was obtained using the averaged relative coordinate of the tracking points using a digital camera (DSC‐RX100M7, Sony) at a rate of 60 frames s^−1^ and processed by commercial software (ProAnalyst Motion Analysis Software, Xcitex). The tilting angle was also evaluated relating the coordinates of the tracking points to the bottom floor. 5 tracking repeats were conducted, and their data were accumulated.

## Conflict of Interest

The authors declare no conflict of interest.

## Author Contributions

S.Y., S.K., and S.L. contributed to conceptualization; S.Y., S.K., J.K., and H.P. worked on methodology; S.Y. and S.K. conducted the investigation and visualization; Y.C. acquired funding, administered the project, and supervised the work; S.Y. prepared the original draft; and S.Y., S.K., J.K., H.P., and Y.C. contributed to review and editing.

## Supporting information



Supporting Information

Supplementary Movie 1‐7

## Data Availability

The data that support the findings of this study are available from the corresponding author upon reasonable request.
