# Supplementary material for: Foldable Soft Leg‐Assisted Wheel Robot
Source: Adv Sci (Weinh). 2025 Oct 6;12(48):e12435. doi: 10.1002/advs.202512435 (PMC12752620; doi:10.1002/advs.202512435)
Supplement: Supplementary file 1 — Supporting Information [file ADVS-12-e12435-s001.pdf]

Supporting Information

Foldable Soft Leg-Assisted Wheel Robot

*Seunghoon Yoo, Sohyun Kim, Joohyeon Kang, Hyunjun Park, Seokjun Lee, and Youngsu Cha**

Email: ys02@korea.ac.kr

The PDF file includes:

Supplementary Note S1 to S4

Figure S1 to S16

Supplementary Table S1 to S6

Other Supporting Information includes the following:

Movie S1 to S7

Supplementary Note

Note S1. Description of the simulation of the FoMs.

A finite element analysis (FEA) was conducted to characterize the mechanical responses of the FoMs on deformation. The simulation was conducted using COMSOL Multiphysics 6.0. The FoMs with different number of levels were set in three space dimensions and solid mechanics physics interface. The CAD geometries were imported from a 3D modeling software (Fusion 360, Autodesk). The layers and hinges were selected for hexahedral meshing, which is advantageous for the material under large deformation.[1] The components were modeled as a linear elastic material with the material parameters in Supplementary Table S1. The end of each structure was constrained as a fixed point, and the other components were free to move.

In the simulation, the relation between the restoring force and axial displacement was studied. Using static analysis, the study performed stepwise solutions over a range of structural displacements. The top layer was moved downward uniformly with an interval of 0.1 mm. The maximum displacement was based on the overall distance between the layers of the FoMs. The performance of the FoM was demonstrated by varying the length of the hinge L. The value was between 4 π to 16 π mm with an interval of 2 π mm using the parametric sweep setup. The restoring force was calculated through the reaction force from the top layers of the FoMs.

The bending angle of the FoMs was also simulated. One edge of the top layer was moved downward in steps of 0.1 mm. Since the simulation continued until the contact of the hinges between the adjacent FoMs, the maximum displacement was obtained when the edge of the top layer ceased to descend and was blocked by the hinges on bending. The angle was calculated based on the relative coordinates of the probes located on the four vertices of the top layer. The restoring force of the FoMs on bending configuration was also calculated by investigating the reaction force on the top layer. Furthermore, the velocity of the tip in the FoMs was also calculated using the relative coordinates and discrete time interval (4.7 ms) based on the frame of the COMSOL.

Note S2. Simplified dynamics model of the FoSLAW.

Figure S9 shows a schematic representation of the FoSLAW. We assume that the FoSLAW simply consists of three centers of mass m_1, m_2, and m_3, which are the points to control in position z_1, z_2, and z_3 on the z-axis. Each m_1 and m_2 contains the two FoMs, whereas the m_3 is the total mass of the FoSLAW except the FoMs. We model the activation of the FoM motors as external loads $F_{1,i}^{j}$ and $F_{2,i}^{j}$ applied to the FoMs. The subscript *i* indicates the types of motion among tilting (*T*) and lifting (*L*), whereas the superscript *j* is the viewpoint side (*S*) and front (*F*). Then, the mechanical response of the FoMs is considered as spring and damper elements *k*(z) and c_{1-4}, capturing the stiffness and dissipative effects from the base foldable materials, respectively.[2, 3] Herein, several researchers employed a third-order polynomial for spring analogy, identifying nonlinear potential profiles of the foldable structures. Motivated by the modeling approaches, the *k*(z) is defined as[4, 5]

$$k(z) = k_1 z + k_2 z^2 + k_3 z^3 \tag{S1}$$

where k_1, k_2, and k_3 represent nonlinear stiffness coefficients characterizing the force-displacement profile of the FoM.

The FoSLAW begins with zero speed and height in the initial condition. We can obtain the resultant forces in the FoSLAW as

$$m_1 \ddot{z}_1 = -k(z_1 - z_3) - k(z_1) - c_1(\dot{z}_1 - \dot{z}_3) - c_1 \dot{z}_1 + F_{1,i}^{j} \tag{S2a}$$

$$m_2 \ddot{z}_2 = -k(z_2 - z_3) - k(z_2) - c_2(\dot{z}_2 - \dot{z}_3) - c_4 \dot{z}_2 + F_{2,i}^{j} \tag{S2b}$$

$$m_3 \ddot{z}_3 = -k(z_3 - z_1) - k(z_3 - z_2) - c_1(\dot{z}_3 - \dot{z}_1) - c_2(\dot{z}_3 - \dot{z}_2) \tag{S2c}$$

where the single and double dots denote the velocity and acceleration of the masses, respectively. Then, the equations of motion can be summarized by a matrix as

$$\begin{bmatrix} m_1 & 0 & 0 \\ 0 & m_2 & 0 \\ 0 & 0 & m_3 \end{bmatrix} \begin{bmatrix} \ddot{z}_1 \\ \ddot{z}_2 \\ \ddot{z}_3 \end{bmatrix} + \begin{bmatrix} c_1 + c_3 & 0 & -c_1 \\ 0 & c_2 + c_4 & -c_2 \\ -c_1 & -c_2 & c_1 + c_2 \end{bmatrix} \begin{bmatrix} \dot{z}_1 \\ \dot{z}_2 \\ \dot{z}_3 \end{bmatrix} + \begin{bmatrix} 2k_1 & 0 & -k_1 \\ 0 & 2k_1 & -k_1 \\ -k_1 & -k_1 & 2k_1 \end{bmatrix} \begin{bmatrix} z_1 \\ z_2 \\ z_3 \end{bmatrix} +$$

$$\begin{bmatrix} k_2(z_1 - z_3)^2 + k_3(z_1 - z_3)^3 + k_2 z_1^2 + k_3 z_1^3 \\ k_2(z_2 - z_3)^2 + k_3(z_2 - z_3)^3 + k_2 z_2^2 + k_3 z_2^3 \\ k_2(z_3 - z_1)^2 + k_3(z_3 - z_1)^3 + k_2(z_3 - z_2)^2 + k_3(z_3 - z_2)^3 \end{bmatrix} = \begin{bmatrix} F_{1,i}^{j} \\ F_{2,i}^{j} \\ 0 \end{bmatrix} \tag{S3}$$

The external loads applied to the FoMs are modeled as first-order responses to a step input in the motor-generated tension. The force components are expressed as

$$F_{1,i}^{j} = A_{1,i}^{j}(1 - e^{-t/T_i}) \quad \text{(S4a)}$$

$$F_{2,i}^{j} = A_{2,i}^{j}(1 - e^{-t/T_i}) \quad \text{(S4b)}$$

where $A_{1,i}^{j}$ and $A_{2,i}^{j}$ represent the steady-state amplitudes of the output normal forces, and T_i denotes the time constant that depends on the types of motion T and L. Since the m_1 and m_2 include the two FoMs for each, the amplitudes are defined as

$$A_{1,i}^{j} = A_{\mathrm{FoLA_A},i}^{j} + A_{\mathrm{FoLA_B},i}^{j} \quad \text{(S5a)}$$

$$A_{2,i}^{j} = A_{\mathrm{FoLA_C},i}^{j} + A_{\mathrm{FoLA_D},i}^{j} \quad \text{(S5b)}$$

where subscript $\mathrm{FoLA_A}$, $\mathrm{FoLA_B}$, $\mathrm{FoLA_C}$, and $\mathrm{FoLA_D}$ express the FoLAs on the robot.

The dynamics model can be modified to represent the FoLAs of the FoSLAW interacting with the platform using equations S2, S4, and S5 by

$$m_1\ddot{z}_1 = -k(z_1 - z_3) - k(z_1) - c_1(\dot{z}_1 - \dot{z}_3) - c_1\dot{z}_1 + F_1^j \quad \text{(S6a)}$$

$$m_2\ddot{z}_2 = -k((z_2 - h) - z_3) - k(z_2 - h) - c_2(\dot{z}_2 - \dot{z}_3) - c_4\dot{z}_2 + F_2^j \quad \text{(S6b)}$$

$$m_3\ddot{z}_3 = -k(z_3 - z_1) - k\big(z_3 - (z_2 - h)\big) - c_1(\dot{z}_3 - \dot{z}_1) - c_2(\dot{z}_3 - \dot{z}_2) \quad \text{(S6c)}$$

$$F_1^j = A_1^j(1 - e^{-t/T_j}) \quad \text{(S6d)}$$

$$F_2^j = A_2^j(1 - e^{-t/T_j}) \quad \text{(S6e)}$$

$$A_1^j = A_{FoLA_A}^j + A_{FoLA_B}^j \quad \text{(S6f)}$$

$$A_2^j = A_{FoLA_C}^j + A_{FoLA_D}^j \quad \text{(S6g)}$$

where h is the height of the platform, and the superscript j is the contraction of the $\mathrm{FoLA_{A,B}}$ (C) and the expansion of the $\mathrm{FoLA_{C,D}}$ (E). In addition, initial conditions of z_1^0 and z_2^0 are considered in the expansion of the $\mathrm{FoLA_{C,D}}$ of the FoSLAW.

The axial and angular displacement u^j and ϕ^j of the prototype can be achieved by

$$\phi^j = \tan^{-1}\left(\frac{z_1 - z_2}{d^j}\right) \quad \text{(S7a)}$$

$$u^j = \frac{d^{j,2}z_1 + d^{j,1}z_2}{d^{j,1} + d^{j,2}} + z_3 \quad \text{(S7b)}$$

where d^j is the distance between the FoLAs in the front and side view. Also, $d^{j,1}$ and $d^{j,2}$ indicate the distances from m_3 to m_1 and m_2, respectively. The detailed values for the parameters are listed in Supplementary Table S3.

Note S3. Equation of energy efficiency and cost of transport (CoT).

Energy efficiency n_w of wheel mode can be calculated as

$$n_w = \frac{mgd^w}{W} = \frac{mgd^w}{W_{FM} + W_{WM}} \quad \text{(S8)}$$

where W_{FM} and W_{WM} represent the energy consumption of the FoM motors and the wheel motors, respectively. m is the robot total mass, g is the gravitational acceleration, and d^w is the total cruise distance of the robot in interest. Herein, the W_{FM} and W_{WM} can be expressed using the electric work done in the motor circuits as

$$W_{FM} = \sum_{i=1}^{8} \int V_F^i(t) I_F^i(t)\, dt \quad \text{(S9a)}$$

$$W_{WM} = \sum_{i=1}^{4} \int V_W^i(t) I_W^i(t)\, dt \quad \text{(S9b)}$$

where V_F^i and V_W^i represent the voltages applied to the i-th FoM motor and wheel motor, respectively, while, I_F^i and I_W^i are the corresponding currents drawn by the i-th FoM motor and wheel motor, respectively. Equation S8 could be converted considering the integration in discrete time measurement systems as

$$W_{FM} = \sum_{i=1}^{8} \sum_{m=1}^{n} V_F^i(t_m) I_F^i(t_m) \delta t_m \quad \text{(S10a)}$$

$$W_{WM} = \sum_{i=1}^{4} \sum_{m=1}^{n} V_W^i(t_m) I_W^i(t_m) \delta t_m \quad \text{(S10b)}$$

where δt_m is the time interval at the time of interest t_n.

The cost of transport (CoT) of the robot during platform stepping also can be given by[6, 7]

$$\mathrm{CoT} = \frac{W}{mgd} = \frac{W_{FM} + W_{WM}}{mgd} \quad \text{(S11)}$$

The effective energy efficiency η_P of the FoSLAW interacting with the platform also can be given by

$$\eta_P = \frac{mgd^P}{W^P} = \frac{mgd^P}{W_{FM}^P + W_{WM}^P} \quad \text{(S12)}$$

where d^P, W_{FM}^P, and W_{WM}^P represent the displacement of the FoSLAW, energy consumption of the FoM and wheel motors from $FoLA_{A,B}$ expansion to $FoLA_{C,D}$ contraction. The detailed parameters are listed in Supplementary Table S5.

Note S4. Platform stepping scenario.

Platform stepping of the FoSLAW is simply explained with the following steps:

1. Front wheels lift: Expand the front FoLAs ($FoLA_A$, $FoLA_B$) to tilt the FoSLAW backward, lifting the front wheels (W_a, W_b) off the ground. This prepares the front FoLAs for stepping onto the platform.
2. Left front FoLA step: Bend the left front FoLA ($FoLA_A$) and drive the rear wheels (W_c, W_d). This causes the FoSLAW to rotate around the right front FoLA ($FoLA_B$), placing the left front FoLA ($FoLA_A$) above the platform.
3. Right front FoLA step: Expand the left front FoLA ($FoLA_A$) to make contact with the platform. Then, bend the right front FoLA ($FoLA_B$) and drive the rear wheels (W_c, W_d) to rotate the body around the left front FoLA ($FoLA_A$). As a result, both front FoLAs ($FoLA_A$, $FoLA_B$) are now positioned on the platform.
4. Front wheels placement: Contract the front FoLAs ($FoLA_A$, $FoLA_B$) so that the front wheels are in contact with the platform surface. Drive the rear wheels (W_c, W_d) to move the rear part of the FoSLAW closer to the platform.
5. Rear wheel lift: Expand the rear FoLAs ($FoLA_C$, $FoLA_D$) to tilt the body forward so that the rear wheels (W_c, W_d) are lifted off the ground, preparing for the rear step-up motion.
6. Left rear FoLA step: With the left rear FoLA ($FoLA_C$) bending, drive the front wheels (W_a, W_b) to rotate the body around the right rear FoLA ($FoLA_D$). This action will raise the left rear FoLA ($FoLA_C$) above the platform.
7. Right rear FoLA step: Expand the left rear FoLA ($FoLA_C$) onto the platform, bend the right rear FoLA ($FoLA_D$), and drive the front wheels (W_a, W_b) to rotate the FoSLAW around the left rear FoLA ($FoLA_C$). This places the right rear FoLA ($FoLA_D$) above the platform as well.

Platform exit: Contract the rear FoLAs ($FoLA_C$, $FoLA_D$), then drive all the wheels (W_{a-d}) to exit the platform completely.

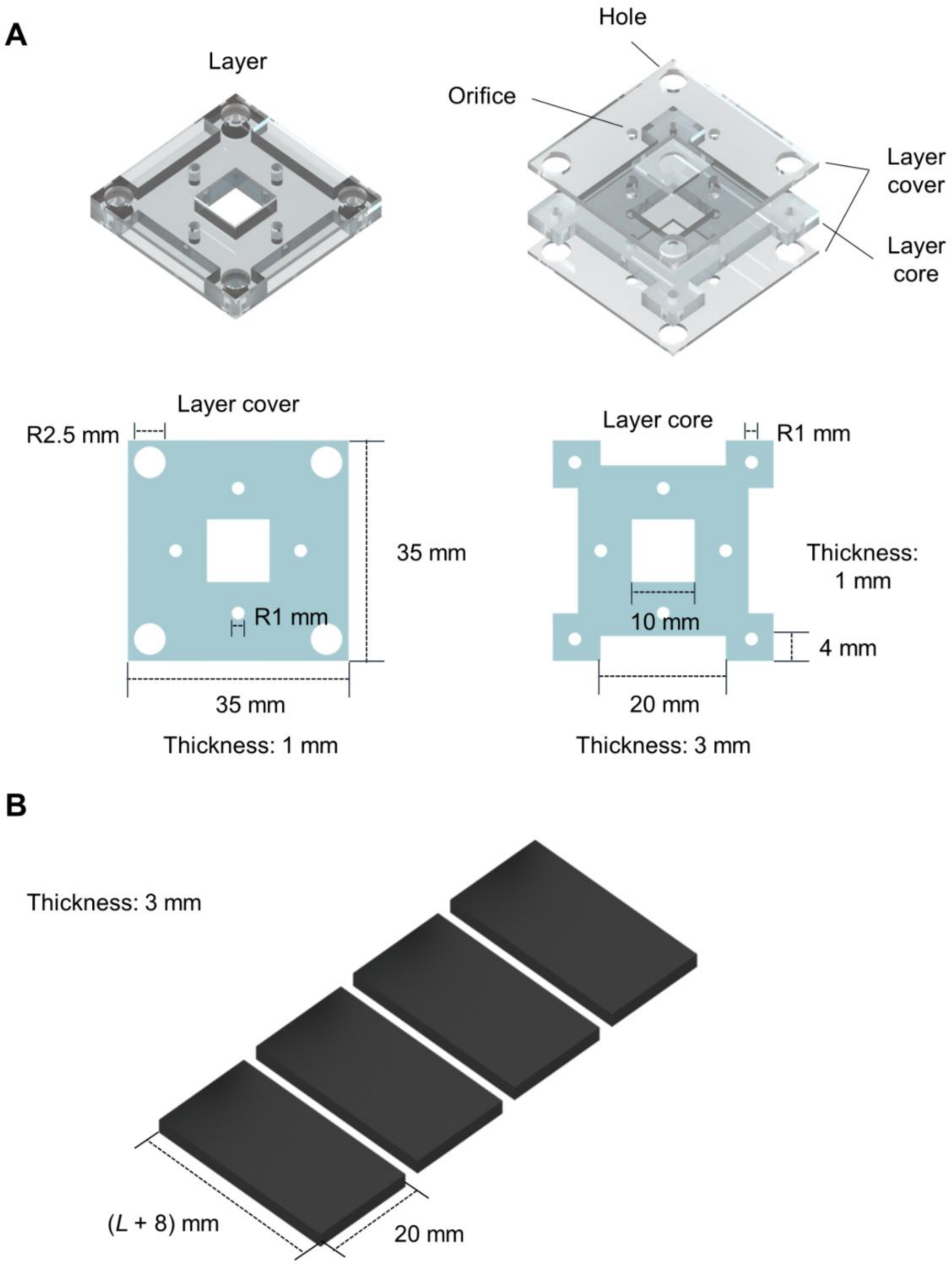

Figure S1. The geometric parameters of the FoM. A) Configuration and geometry dimension of the layer. B) Geometry dimension of the hinge.

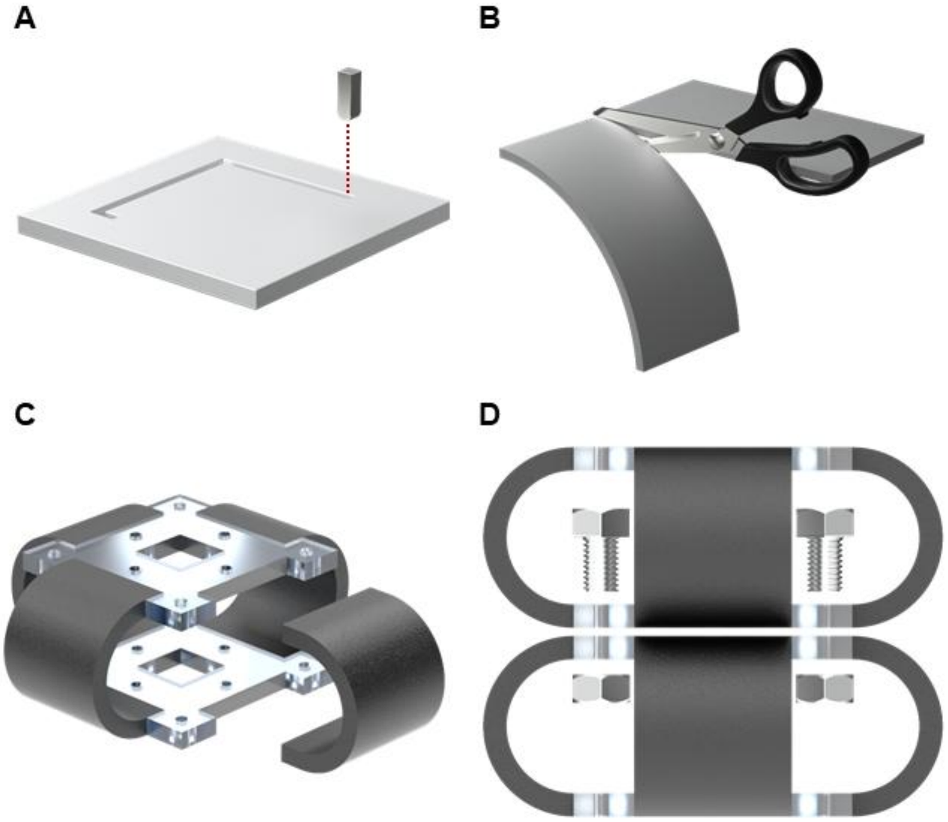

Figure S2. The fabrication process of the FoMs. A) Cutting the acrylic board into a layer cover and core using laser-machining technology using computer-aided design (CAD). B) Cutting a rubber pad using a straw cutter into a rectangle (40 mm × 58 mm). C) Bonding the layers and hinges for the FoM. D) Assembling the levels to complete the FoMs by interlocking.

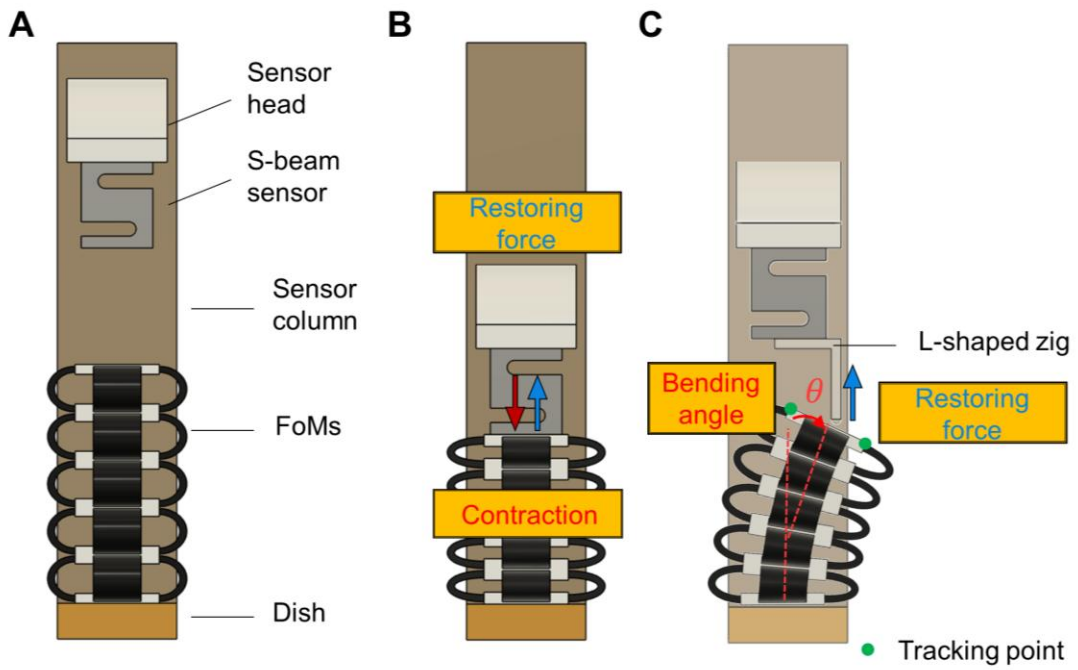

Figure S3. The measurement process of the FoMs. A) Schematic of a measurement system including the force sensor and FoMs. The schematic of the restoring force and bending angle measurement in which the FoMs is B) contracted linearly and C) bent by the L-shaped zig.

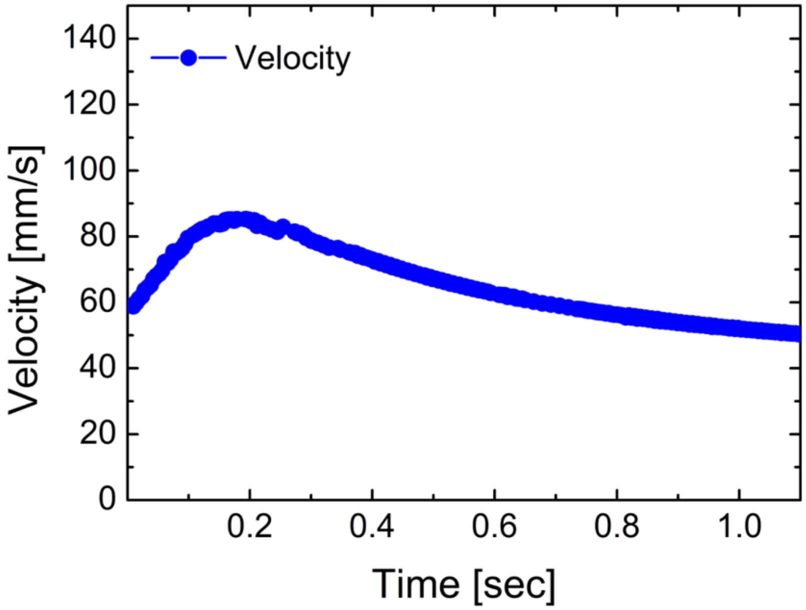

Figure S4. Velocity of the tip during the FoM on directional bending in simulation.

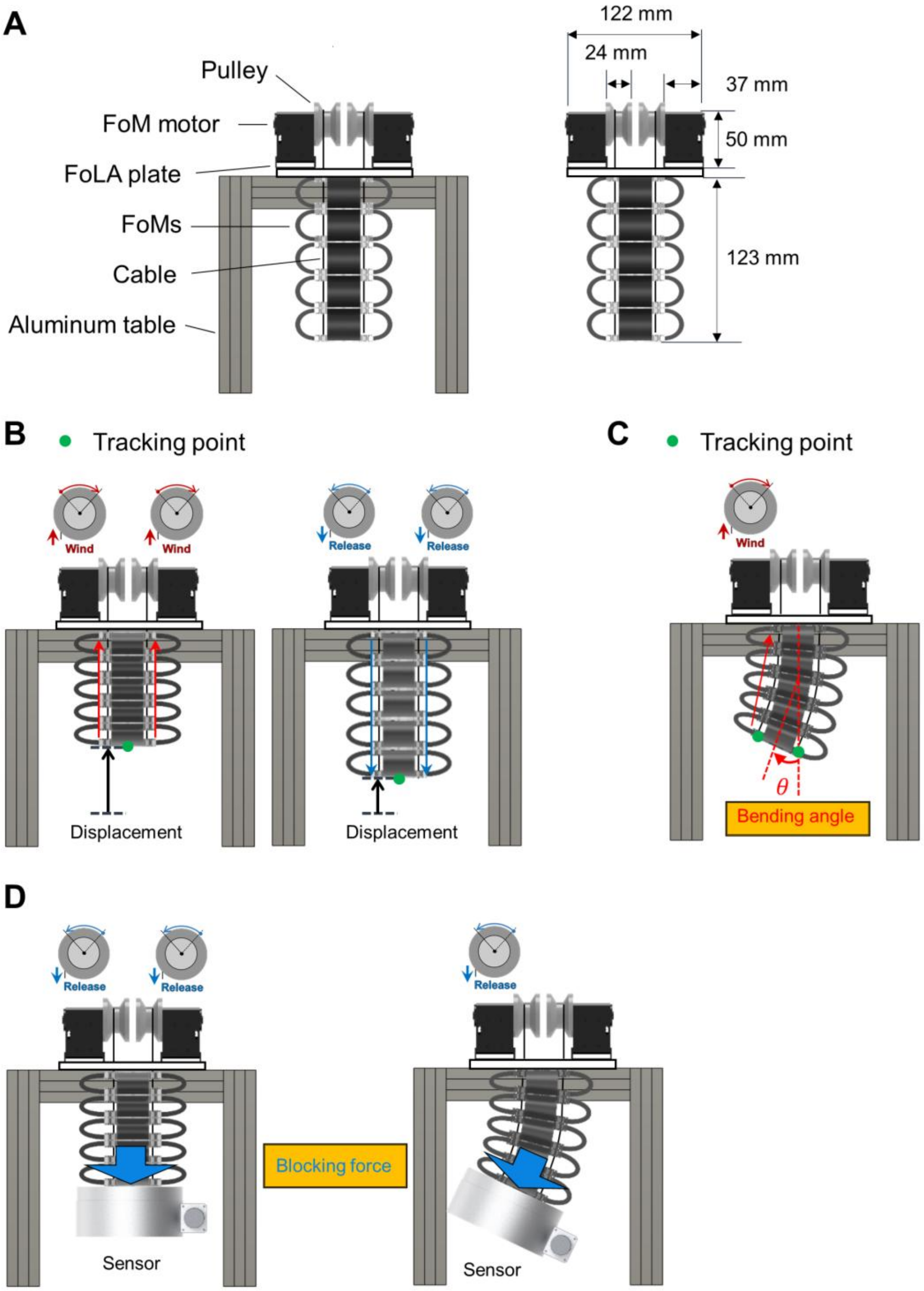

Figure S5. The measurement process of mechanical performances from the FoLA. A) Configuration and dimension of the FoLA. The schematic of measurement for B) linear displacement and C) bending angle of the FoLA. D) The schematic of blocking force measurement from the FoLA in response to linear and bending deformation.

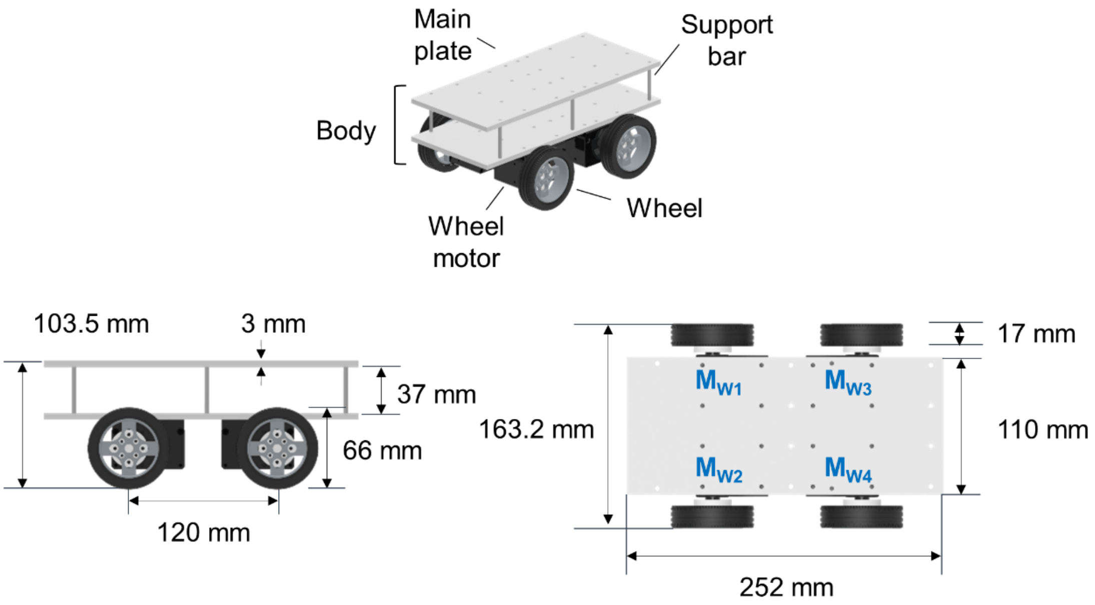

Figure S6. 3D model and dimensions of the Wheel Robot (WR).

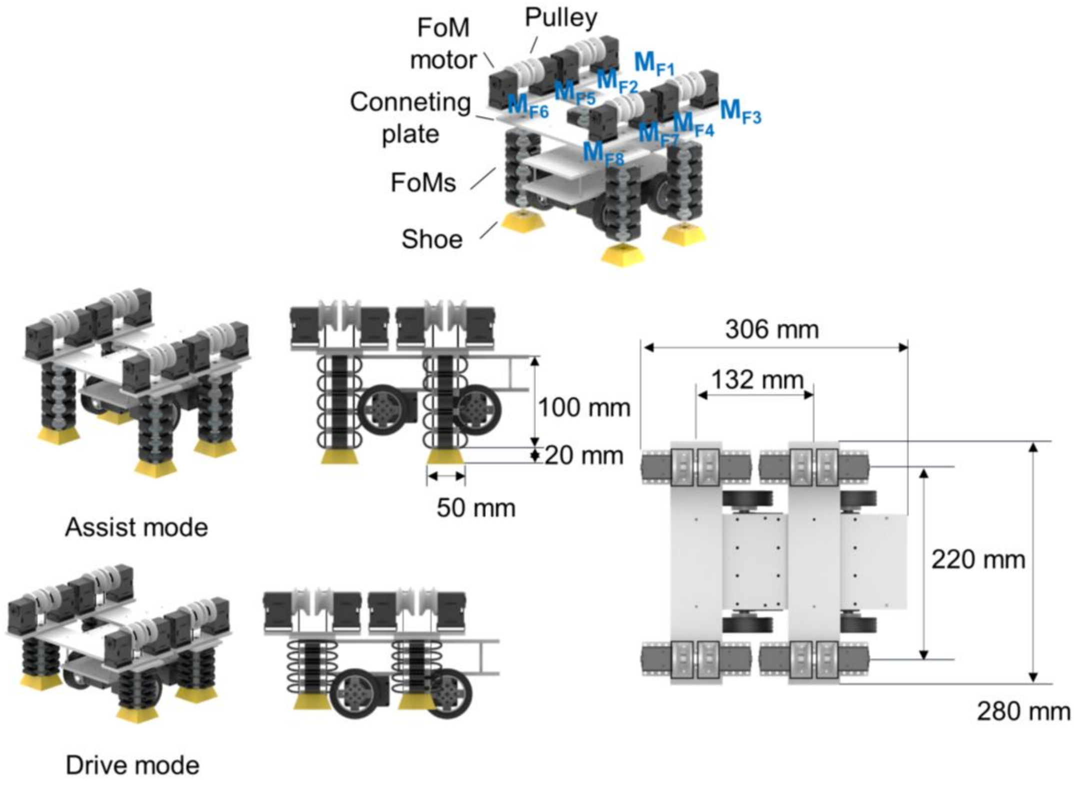

Figure S7. 3D model and dimensions of the FoSLAW.

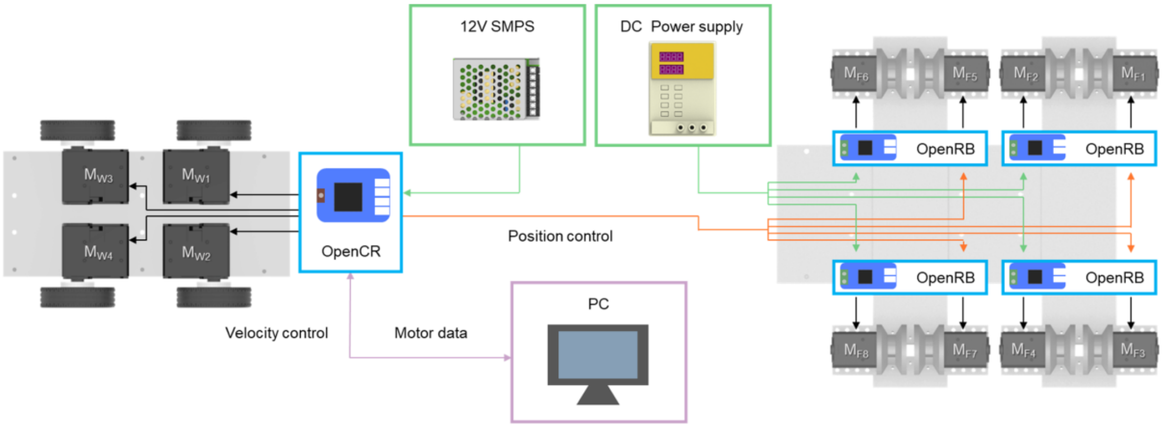

Figure S8. Schematic motor control diagram of the FoSLAW.

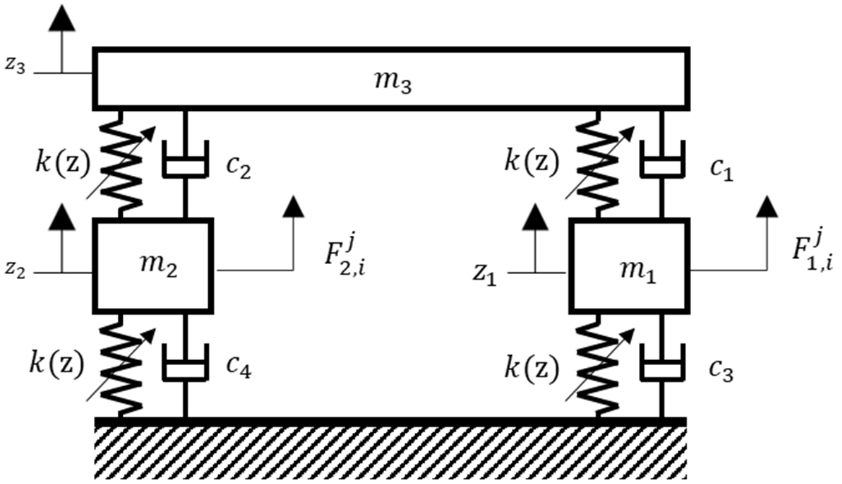

Figure S9. Three degrees of freedom spring-mass-damper system analogy of the FoSLAW.

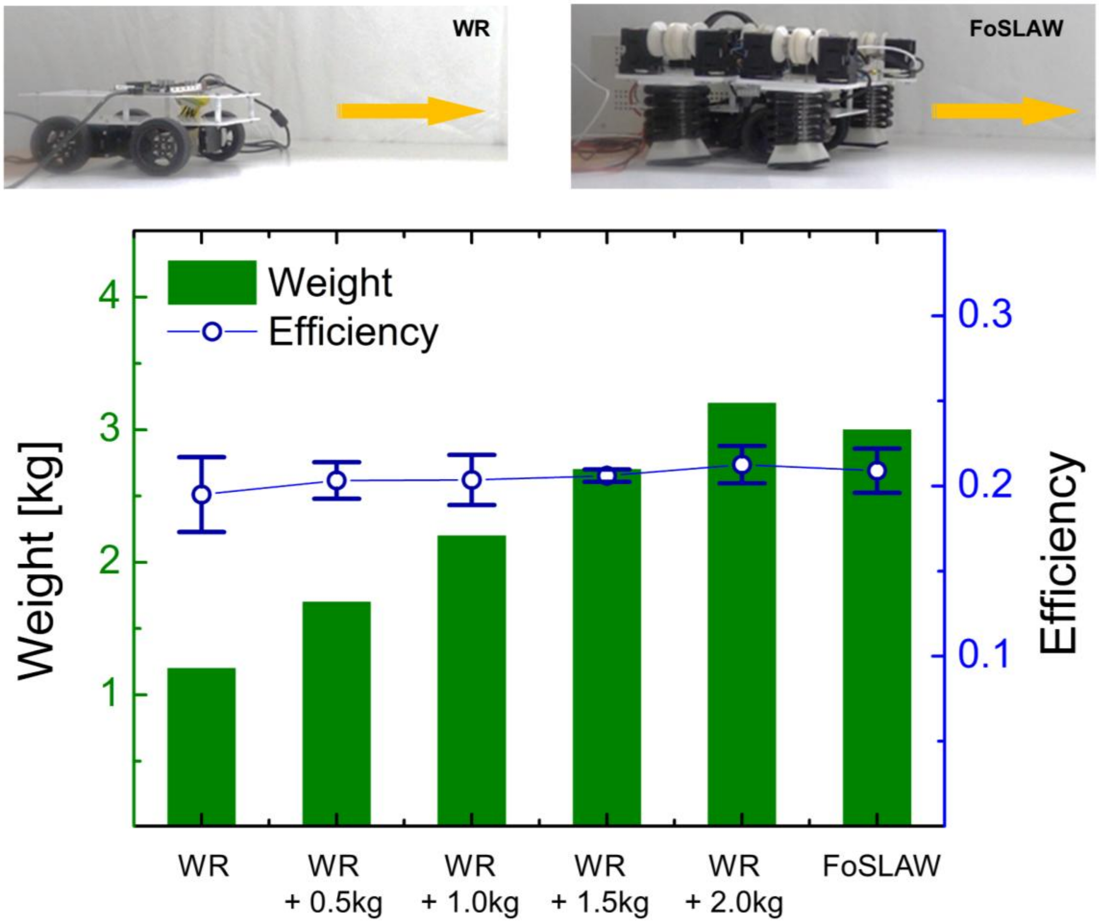

Figure S10. Wheel mode experiment and efficiency with additional weight on the robots.

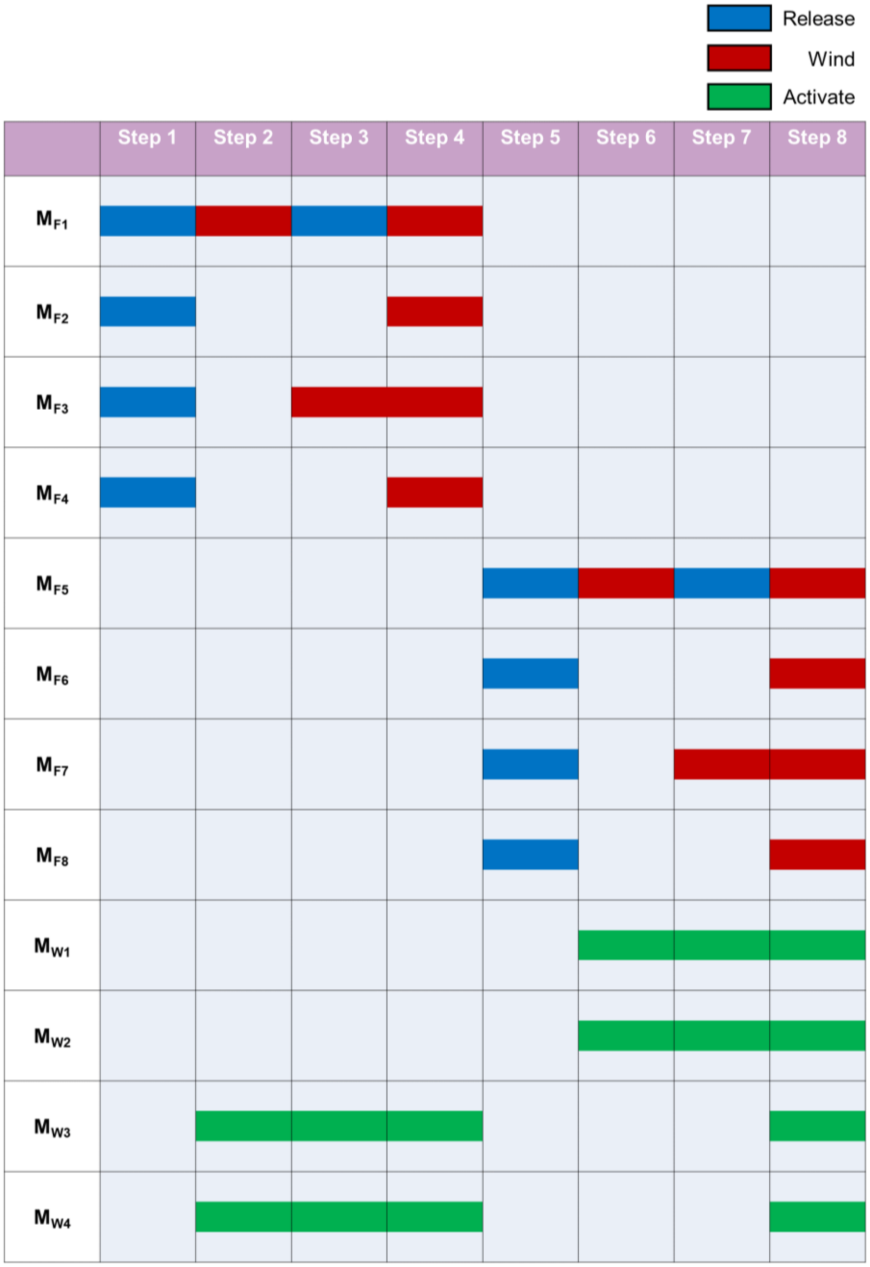

Figure S11. Motor control sequence of the FoSLAW in platform stepping.

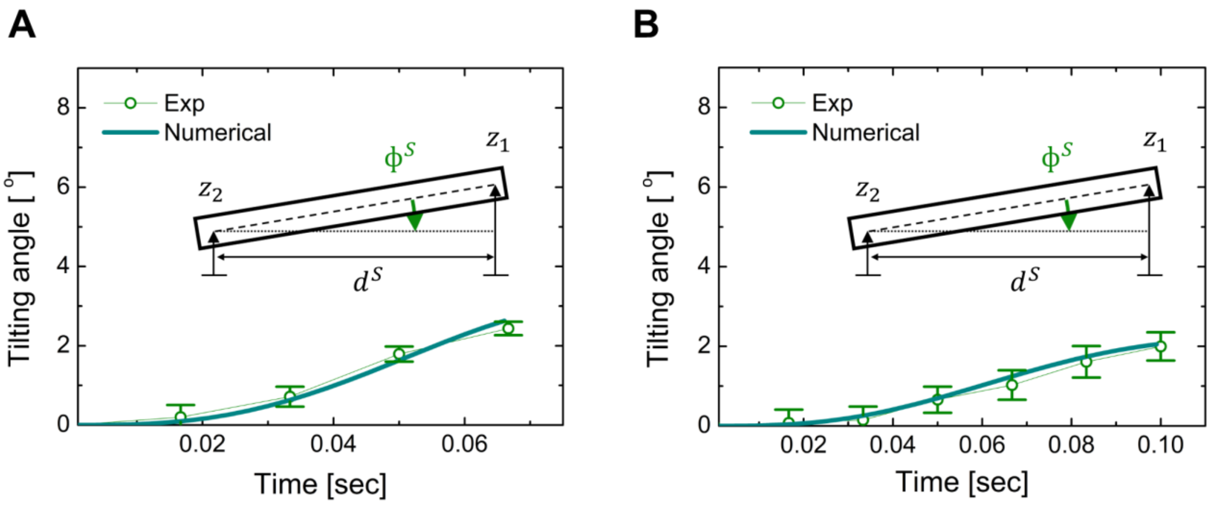

Figure S12. Experimental and numerical results of the tilting angle by A) $\mathbf{FoLA_{A,B}}$ contraction and B) $\mathbf{FoLA_{C,D}}$ expansion.

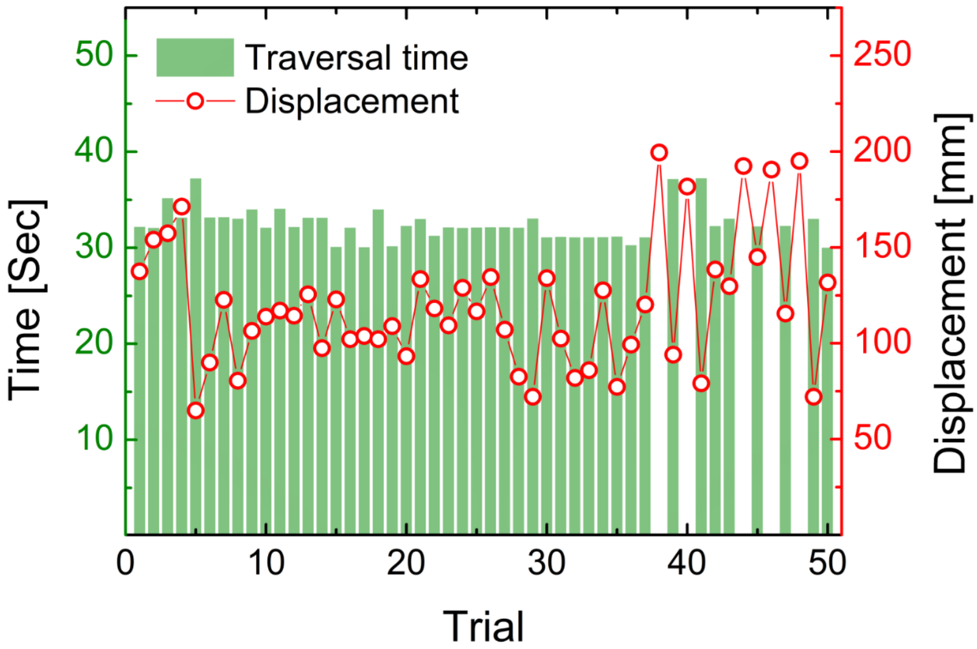

Figure S13. Experimental results of the repeated platform stepping.

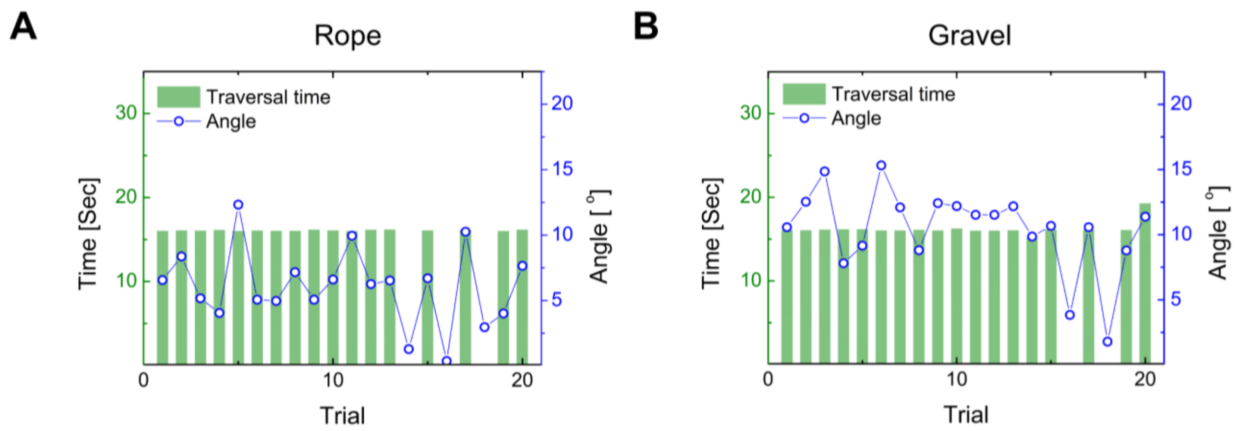

Figure S14. Experimental details for traversing A) flexible rope and B) Gravel terrain.

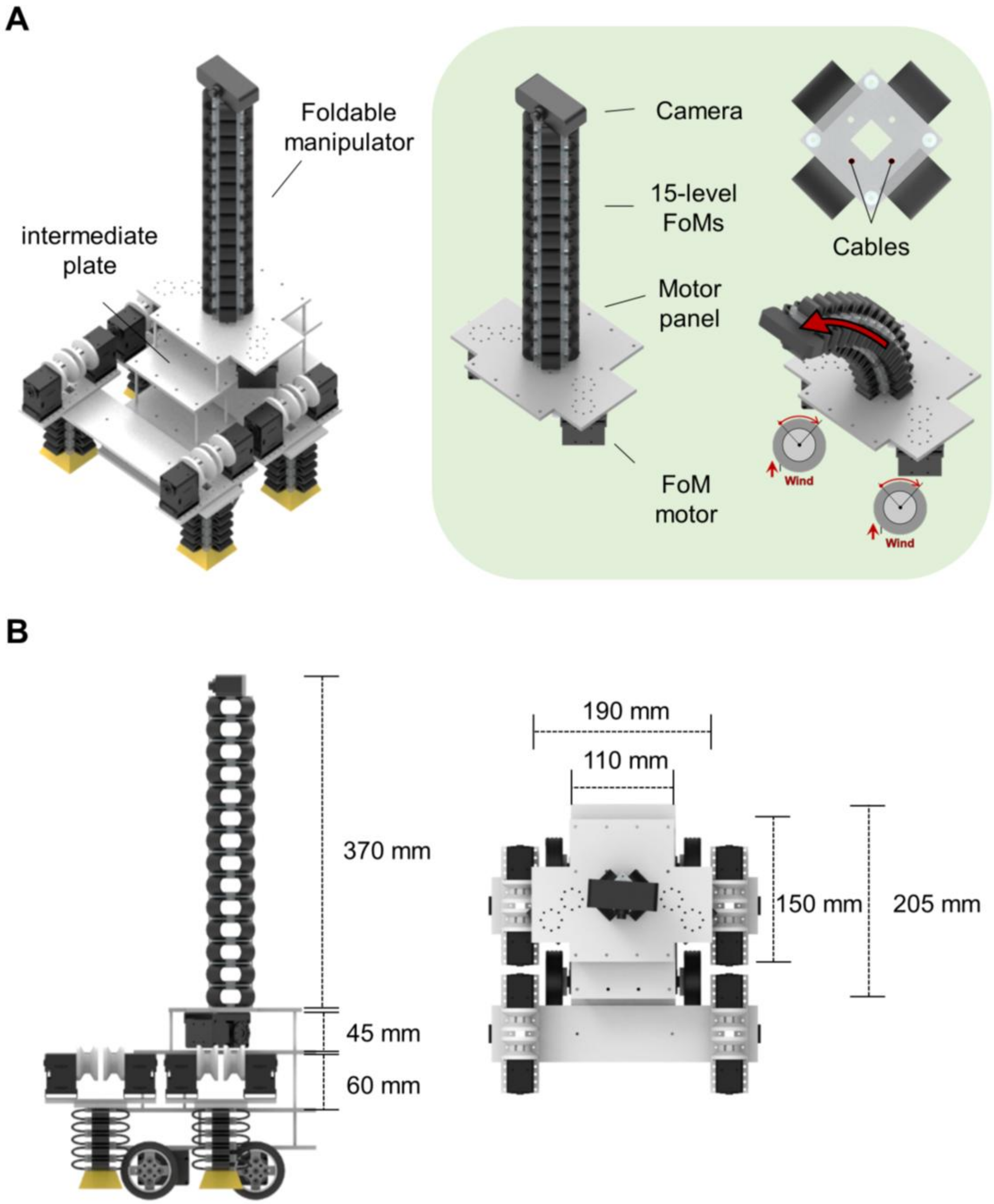

Figure S15. 3D model of the manipulator-equipped FoSLAW. A) Configuration and activation schematic of the foldable manipulator. B) Geometry dimension of the foldable manipulator on the mobile robot.

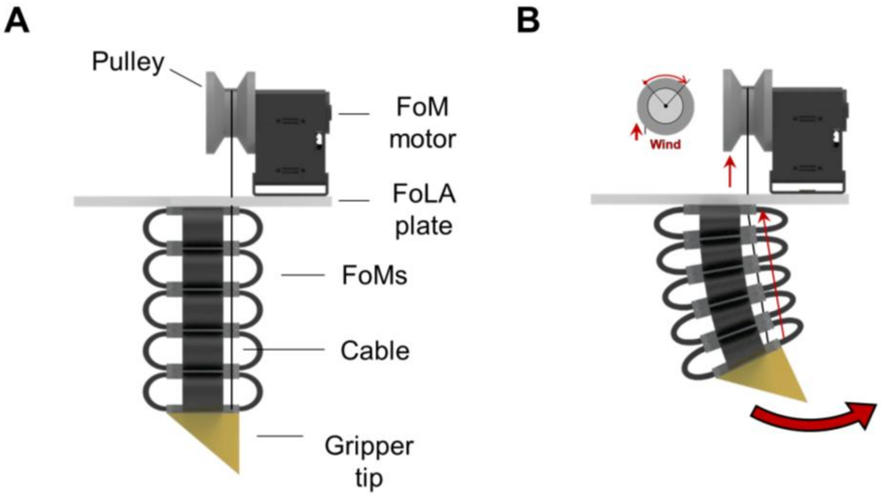

Figure S16. 3D model of the gripper finger. A) Configuration and B) activation of the gripper finger.

Supplementary Tables

Supplementary Table S1. Material parameters of the FEA simulation.

Material properties	Rubber	Acrylic
Young's modulus [MPa]	4.6	3200
Density [$\mathbf{g}/\mathbf{cm^3}$]	1.69	1.19
Poisson's ratio	0.49	0.35

Supplementary Table S2. Details for frequency tests of the FoLA.

Test type			Contraction	Bending
Value type			Peak-to-peak displacement [mm]	Bending angle [°]
Frequency	0.6 Hz	Average	58.48	57.65
		Standard deviation	0.01	0.23
	0.75 Hz	Average	56.93	57.90
		Standard deviation	0.02	0.15
	0.9 Hz	Average	58.38	57.99
		Standard deviation	0.02	0.12
	1.05 Hz	Average	57.60	58.80
		Standard deviation	0.02	0.16
	1.2 Hz	Average	58.14	55.46
		Standard deviation	0.73	0.90

Supplementary Table S3. Parameters of the dynamics model.

Symbol	Value	Symbol	Value	Symbol	Value	Symbol	Value
$m_{1,2}$	0.4 kg	m_3	2.2 kg	k_1	427 N/m	k_2	-7760 N/m^2
k_3	78900 N/m^3	c_{1-4}	1.61 Ns/m	$A^S_{\mathrm{FoLA_A},T}$	8.5 N	$A^S_{\mathrm{FoLA_B},T}$	8.5 N
$A^S_{FoLA_C,T}$	0 N	$A^S_{FoLA_D,T}$	0 N	$A^S_{FoLA_A,L}$	5 N	$A^S_{\mathrm{FoLA_B},L}$	5 N
$A^S_{FoLA_C,L}$	1.5 N	$A^S_{\mathrm{FoLA_D},L}$	1.5 N	$A^F_{\mathrm{FoLA_A},T}$	8.5 N	$A^F_{\mathrm{FoLA_B},T}$	0 N
$A^F_{\mathrm{FoLA_C},T}$	7.2 N	$A^F_{\mathrm{FoLA_D},T}$	0 N	$A^F_{\mathrm{FoLA_A},L}$	5 N	$A^F_{\mathrm{FoLA_B},L}$	5 N
$A^F_{\mathrm{FoLA_C},L}$	1.5 N	$A^F_{\mathrm{FoLA_D},L}$	1.5 N	$A^C_{\mathrm{FoLA_A}}$	-8.5 N	$A^C_{\mathrm{FoLA_B}}$	-8.5 N
$A^C_{\mathrm{FoLA_C}}$	0 N	$A^C_{\mathrm{FoLA_D}}$	0 N	$A^E_{\mathrm{FoLA_A}}$	0 N	$A^E_{\mathrm{FoLA_B}}$	0 N
$A^E_{\mathrm{FoLA_C}}$	5 N	$A^E_{\mathrm{FoLA_D}}$	5 N	T_T	3.7 s	T_L	2.0 s
T_C	0.066 s	T_E	0.1 s	d^S	132 mm	d^F	220 mm
$d^{S,1}$	32 mm	$d^{F,1}$	110 mm	$d^{S,2}$	100 mm	$d^{F,2}$	110 mm
h	10 mm	z_1^0	-0.7 mm	z_2^0	13.6 mm		

Supplementary Table S4. Details for traversing complex terrain.

Terrain		Platform	Rope	Gravel
Success rate		90 %	85 %	90 %
Traversal time	Average	31.1 s	16.1 s	16.22 s
	Standard deviation	6.837 s	0.061 s	0.796 s
Displacement	Average	115 mm	-	-
	Standard deviation	28.83 mm	-	-
Angle	Average	-	6.86°	11.24°
	Standard deviation	-	2.28°	1.98°

Supplementary Table S5. Parameters of the cost of transport (CoT) and efficiency $\boldsymbol{\eta_p}$.

Symbol	Value	Symbol	Value	Symbol	Value
m	3 kg	g	9.81 m/s^2	d	255 mm
d^w	900 mm	d^p	230 mm	δt_m	0.05 s
t_n	27 s				

Supplementary Table S6. Benchmarking table comparing FoSLAW against existing robots.

	FoSLAW	Leg-wheel robots[8-10]			Retractable leg robots[11-13]			Soft foldable robots[14-16]		
		[8]	[9]	[10]	[11]	[12]	[13]	[14]	[15]	[16]
Body height [mm]	120	200	460	327	316	275	295	4	55	33
Weight [kg]	3	9.6	7	6.1	unknown	0.5	5.5	unknown	1.35	0.008
Obstacle type	Complex terrains	Simple terrains			Simple terrains			Unstructured environments		
Mechanism	Soft leg-wheel hybrid	Leg-wheel hybrids			Transformable wheels			Foldable structures		
Locomotion type	Leg-wheel synergy	Leg-wheel switching			Adjustable wheel			Morphing or Crawling		

Movie S1.

Actuation experiments of the FoLA

Movie S2.

Maneuver experiment of the FoSLAW to traverse a convex terrain

Movie S3.

Motion experiment of platform stepping

Movie S4.

Traversal experiment of gravel terrain

Movie S5.

Traversal experiment of rope

Movie S6.

Foldable manipulator application

Movie S7.

Object grasping experiments

References

[1] L. Ding, L. Niu, Y. Su, et al., "Dynamic finite element modeling and simulation of soft robots," *Chinese journal of mechanical engineering* **2022**, *35* (1), 24.
[2] J. Kim, E. Im, Y. Lee, Y. Cha, "Quadrupedal robot with tendon-driven origami legs," *Sensors and Actuators A: Physical* **2024**, *378*, 115769.
[3] A. S. Dalaq, M. F. Daqaq, "Experimentally-validated computational modeling and characterization of the quasi-static behavior of functional 3D-printed origami-inspired springs," *Materials & Design* **2022**, *216*, 110541.
[4] H. Fang, S. Li, H. Ji, K. Wang, "Dynamics of a bistable Miura-origami structure," *Physical Review E* **2017**, *95* (5), 052211.
[5] Z. Liu, X. Zhang, K.-W. Wang, J. Xu, H. Fang, "Data-driven modeling of multi-stable origami structures: Extracting the global governing equation and exploring the complex dynamics," *Mechanical Systems and Signal Processing* **2024**, *220*, 111659.
[6] N. Kottege, C. Parkinson, P. Moghadam, A. Elfes, S. P. Singh, "Energetics-informed hexapod gait transitions across terrains," in *2015 IEEE Int. Conf. Robot. Autom.* IEEE, **2015**, 5140-5147.
[7] M. Prágr, P. Čížek, J. Faigl, "Cost of transport estimation for legged robot based on terrain features inference from aerial scan," in *2018 IEEE/RSJ Int. Conf. Intell. Robots Syst* IEEE, **2018**, 1745-1750.
[8] H. Xia, Y. He, H. Lei, Q. Sun, "Jump Planning and Airborne Attitude Control of Bipedal Wheel-Legged Robots," *IEEE Robotics and Automation Letters* **2025**.
[9] F. Iotti, A. Ranjan, F. Angelini, M. Garabini, "OmniQuad: A wheeled-legged hybrid robot with omnidirectional wheels," *Mechanism and Machine Theory* **2025**, *214*, 106125.

[10] J. Ma, M. Zhu, T. Zhang, E. Capello, X. Yue, "Flexibility and granular terrain adaptability of a linkage-based wheel-legged robot: LinkWheg," *IEEE/ASME Transactions on Mechatronics* **2025**.
[11] L. Han, F. Ding, L. Zhao, et al., "Design and motion analysis of a coal mine robot with variable wheel diameter," *Scientific Reports* **2025**, *15* (1), 6497.
[12] C. Zheng, S. Sane, K. Lee, V. Kalyanram, K. Lee, "a-WaLTR: Adaptive wheel-and-leg transformable robot for versatile multiterrain locomotion," *IEEE Transactions on Robotics* **2022**, *39* (2), 941.
[13] R. Cao, J. Gu, C. Yu, A. Rosendo, "Omniwheg: An omnidirectional wheel-leg transformable robot," in *2022 IEEE/RSJ International Conference on Intelligent Robots and Systems (IROS)* IEEE, **2022**, 5626-5631.
[14] Q. Ze, S. Wu, J. Dai, et al., "Spinning-enabled wireless amphibious origami millirobot," *Nature communications* **2022**, *13* (1), 3118.
[15] J. Liu, Z. Pang, Z. Li, et al., "An origami-wheeled robot with variable width and enhanced sand walking versatility," *Thin-Walled Structures* **2025**, *206*, 112645.
[16] X. Ren, Y. Huan, M. Cianchetti, et al., "Soft Crawling Robot With a Dual-Morphing Origami Configuration," *IEEE Transactions on Medical Robotics and Bionics* **2024**.
